# Oral Manifestations in the Post COVID‐19 Condition: A Systematic Review With Meta‐Analysis

**DOI:** 10.1002/rmv.70057

**Published:** 2025-07-15

**Authors:** Letícia Simeoni Avais, Elis Carolina Pacheco, Luisa Pereira de Oliveira Zanetti Gomes, Márcia Helena Baldani, Camila Marinelli Martins, Eliseu Alves Waldman, Jean Paul J. Gonzalez, Tomoko Y. Steen, Pollyanna Kássia de Oliveira Borges

**Affiliations:** ^1^ Department of Microbiology and Immunology Georgetown University School of Medicine Washington District of Columbia USA; ^2^ Graduate Program in Dentistry, State University of Ponta Grossa (UEPG) Ponta Grossa Brazil; ^3^ University of Joinville (UNIVILLE) Joinville Brazil; ^4^ Department of Dentistry State University of Ponta Grossa Ponta Grossa Brazil; ^5^ Department of Medicine State University of Ponta Grossa Ponta Grossa Brazil; ^6^ Department of Epidemiology College of Public Health University of São Paulo São Paulo Brazil; ^7^ Department of Public Health State University of Ponta Grossa Ponta Grossa Brazil

**Keywords:** long COVID, oral health, oral manifestations, post‐acute COVID‐19 syndrome

## Abstract

Post‐COVID‐19 condition, or Long COVID, is characterised by symptoms persisting or emerging beyond 12 weeks after acute infection. Among over 200 reported symptoms, oral manifestations such as taste loss and dry mouth have been identified. This systematic review reports the frequency and characteristics of these symptoms. Registered in PROSPERO and following PRISMA guidelines, the review included observational studies on COVID‐19‐positive adults presenting oral symptoms in the post‐COVID‐19 condition. A search in six databases (Medline/PubMed, Embase, Web of Science, Cochrane, SCOPUS, and LILACS) was conducted in January 2024. Risk of bias was assessed using Joanna Briggs Institute's critical appraisal tools, and certainty of evidence via GRADE. A meta‐analysis using the inverse variance method estimated oral symptom prevalence. Of 4552 articles, 107 were included. Taste dysfunction persisted in 8% (95% CI 6%–10%) of patients beyond 12 weeks. Combined taste and smell alterations had a prevalence of 17% (95% CI 13%–21%). Less frequent symptoms included hyposalivation, periodontitis, mouth ulcers, tongue mucosal changes, facial tingling, sensitivity in the trigeminal nerve, difficulty swallowing, and lesions in the hard palate. Taste alterations were the most commonly reported symptom, underscoring the need for clinical recognition and appropriate management by oral health professionals. Additionally, the wide range of other oral manifestations highlights the necessity for further research to better understand their prevalence, underlying mechanisms, and clinical implications in post‐COVID‐19 patients.

## 1 Introduction

In 2020, the World Health Organization (WHO) declared a public health emergency caused by Coronavirus disease (COVID‐19) [1, 2]. Transmission of the disease has been found to occur from person to person, by respiratory tract droplets or contact [3]. Individuals may have symptoms such as fever, cough, and fatigue [3, 4, 5]. The acute condition of COVID‐19 shows remission from 2 to 6 weeks [5]. Between 10% and 20% of patients report long‐term manifestations of persistent symptoms, or new symptoms and diseases [6]. This condition is defined by WHO as a post‐COVID‐19 condition and occurs in individuals with a history of SARS‐CoV‐2 infection, usually beginning 3 months after acute COVID‐19 [7].

Although immunisation has drastically impacted the reduction of cases and deaths from acute COVID‐19 [8], post‐COVID‐19 conditions are being described frequently. There are reports of symptoms different from those that occurred in the acute phase [9]. The most cited symptoms are fatigue, difficulty breathing, decreased memory and concentration, and loss of taste and smell [7, 10, 11]. Despite the growing volume of research on post‐COVID‐19 conditions, studies addressing oral manifestations are still scarce, especially regarding symptoms other than loss of smell and taste [12, 13, 14]. In fact, oral symptoms are not frequently described in clinical studies on COVID‐19 [15], and among the few existing studies, taste alterations are the most commonly reported oral manifestation [16].

Oral manifestations will continue to affect individuals with post‐COVID conditions, just like other persistent symptoms. It is essential to deepen our understanding of its symptoms and prevalence while ensuring that oral healthcare professionals are aware of its existence to enhance their clinical practice [17].

To summarise the main current scientific evidence to support clinical dental surgeons and researchers, this study aimed to investigate what are the oral manifestations of the post‐COVID‐19 condition and the frequency that they present.

## 2 Methods

This is a systematic review study, with meta‐analysis. Registered on the PROSPERO platform under number CRD42022336065 and following the PRISMA guidelines [18]. The guiding question of the research was: What are the most frequent oral manifestations of the post‐COVID‐19 condition?

Observational studies (cross‐sectional, cohort, case reports) that provided information on the prevalence or incidence of oral manifestations in the post‐COVID‐19 condition were included.

### 2.1 Eligibility Criteria

Studies were eligible for inclusion if they reported data on the prevalence of oral manifestations in adults (≥ 18 years) with confirmed COVID‐19 and assessed symptoms occurring three months or more after the acute phase of infection, consistent with the WHO definition of post‐COVID‐19 condition. Importantly, studies did not need to include only symptomatic individuals; they were included as long as at least one oral manifestation was evaluated in the total sample. No restrictions were applied regarding publication date or language.

Studies that lacked a clear definition of the post‐COVID‐19 period or provided incomplete data on the prevalence of oral symptoms were excluded. Incomplete data referred to cases where oral symptoms were initially assessed but not reported in the results beyond 3 months or where only overall percentages were presented, without detailed information on symptom persistence.

### 2.2 Information Sources and Search Strategy

The searches were carried out in the Medline, Embase, Web of Science, Cochrane, SCOPUS, and LILACS databases in January/2024.

The search strategy included both controlled vocabulary (Medical Subject Headings—MeSH) and free terms (uncontrolled vocabulary). The search terms were combined using the Boolean operators “AND” and “OR” (see Supporting Information S1). The research question was structured according to the PEO format: Population (P)—individuals with post‐COVID‐19 condition (Long COVID); Exposure (E)—signs and symptoms characterising the post‐COVID‐19 condition; Outcome (O)—frequency of oral manifestations.

### 2.3 Selection Process

The studies were collected from the electronic databases and organised in the *Mendeley* software to manage the list of articles and identify duplicates. After the duplicates were removed, the studies were initially analysed based on the titles and then abstracts. Two researchers independently performed the selection of the studies, and a third solved additional differences, when necessary.

Abstracts without sufficient information were retained for evaluation of the full text. Those who met the eligibility criteria had their full text retrieved and, again, both researchers assessed eligibility.

### 2.4 Data Collection Process and Data Items

One reviewer extracted the data from the included articles and a second reviewer checked the agreement of the studies to resolve interpretative differences. A third reviewer was consulted for consensus.

### 2.5 Effect Measures and Synthesis Methods

The data extracted from the included surveys were organised in an Excel 2016 spreadsheet (Supporting Information S2), according to patient symptomatology, sample number of each study, and number of patients with the symptom.

We also collected the year of publication, geographic location, definition of time used for the post‐COVID‐19 condition, type of study, number of subjects sampled, symptoms of the post‐COVID‐19 condition evaluated, acute COVID‐19 condition in the study patients, conclusion, and limitations of the study (Supporting Information S2).

In case of incomplete data, there was an attempt to contact the corresponding author. When there was no response, the study was excluded. For a better visualisation of the results, the time was standardized in days but always following the WHO definition.

The meta‐analysis models analysed the data from the articles to estimate the frequencies of oral manifestations in patients with post‐COVID‐19 conditions. The number of patients with oral manifestations and the total number of patients evaluated were used. The meta‐analysis method employed was inverse variance for proportions [19].

Two meta‐analyses were performed: (1) alteration of taste and (2) alteration of smell and taste collected concomitantly, because they presented sufficient quantities of studies for meta‐analysis. Analyses were presented with a 95% confidence interval and plotted in Forest plots.

Statistical heterogeneity between studies was assessed using the I^2^ statistic, with higher values indicating greater inconsistency. However, the choice of a random‐effects model was based primarily on anticipated clinical and methodological heterogeneity among studies (such as differences in study populations, diagnostic criteria, and follow‐up periods) which is expected in prevalence studies [20]. All analyses were performed in R 4.1.1 (R core team, 2021) with “meta” package [19].

### 2.6 Study Risk of Bias Assessment

Two reviewers assessed the risk of bias in the included studies. We use the Joanna Briggs Institute's (JBI) critical appraisal tools for prevalence studies, case reports and case series [21] to assist in assessing the trustworthiness, relevance, and results of published papers (Supporting Information S2). Studies were classified as having a high risk of bias when the ‘yes’ score was up to 49%, moderate risk when the score ranged from 50% to 69%, and low risk when it exceeded 70% [15, 21].

### 2.7 Certainty Assessment

We used the Grading of Recommendations Assessment, Development and Evaluation (GRADE) approach to assess certainty. Evaluation was conducted for both outcomes (changes in taste and in taste and/or smell) where meta‐analysis was feasible.

## 3 Results

**FIGURE 1:**
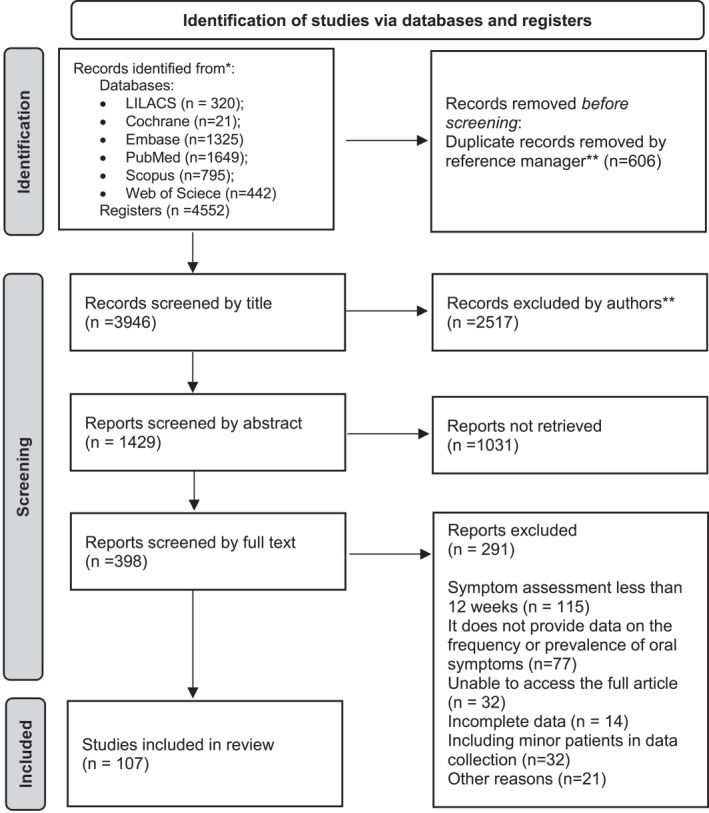
PRISMA flowchart of identifying studies using the search and registration bases of the inclusion strategy for studies on the post‐COVID‐19 condition. *Consider, if feasible to do so, reporting the number of records identified from each database or register searched (rather than the total number across all databases/registers). **If automation tools were used, indicate how many records were excluded by a human and how many were excluded by automation tools.

Four thousand and five hundred and fifty‐two (4552) articles were found, and, after exclusions, 107 studies were analysed (Figure 1). Of these, 105 had information on taste and/or smell alteration, 2 addressed swallowing difficulty and 9 were about oral manifestations (excluding taste alteration).

The number of participants per study varied widely, ranging from case reports with a single individual to large cohort studies, the largest of which included 486,149 participants. Excluding this exceptional study, the sample sizes ranged from 1 to 5963. The reported age of participants ranged from 18 to 87 years. Most studies included adult populations, with age presented as mean, median, or minimum threshold (≥ 18 years). Studies with insufficient demographic detail were noted and categorised accordingly in the data extraction table (Table 1).

**TABLE 1:** Description of studies included in the systematic review, according to type of study, post‐COVID‐19 evaluation time, age of participants, post‐COVID‐19 oral manifestations, and others. Ponta Grossa‐Parana, Brazil: 2024.

Author, year of publication	Country	Study type	Minimum time assessed after the acute phase of COVID‐19**	Minimum time assessed after the acute phase of COVID‐19 (days)***	Number of participants	Age of participants**	Characteristics of COVID‐19 infection	Oral manifestations present
Aranda Rubio et al., 2021 [22]	Spain	Case report	3 months	90 days	1	87	Presented with bilateral COVID‐19 pneumonia	Metallic taste sensation
Asadi‐Pooya et al., 2021 [23]	Iran	Cohort	3–12 months	90–360 days	4681	Mean 52 (± 15)	Included all degrees of the disease	Anosmia and ageusia
Augustin et al., 2021 [24]	Germany	Cohort*	4–7 months	120–210 days	958	Mean 43 (31–54)	Included all degrees of the disease	Anosmia and ageusia
Biadsee et al., 2021 [12]	Israel	Cross‐sectional	8 months	240 days	97	Mean 37.5 (19–74)	Non‐hospitalised	Dysgeusia, dysosmia and xerostomia
Brito‐Zerón et al., 2021 [25]	Spain	Cross‐sectional*	> 12 Weeks	> 84 days	132	Mean 54.8 (± 13.9)	Included all degrees of the disease in patients with primary Sjögren's syndrome	Anosmia and dysgeusia
Fernández‐de‐las‐Peñas et al., 2021 [26]	Spain	Cohort*	7 months	210 days	1142	Mean 61 (± 17)	Included all degrees of the disease	Anosmia and ageusia
Gherlone et al., 2021 [13]	Italy	Cohort	> 3 months	> 90 days	122	Mean 62.5 (53.9–74.1)	Included all degrees of the disease	Dysgeusia, anosmia, xerostomia, tongue abnormalities, salivary gland ectasia, facial tingling, weakness in the masticatory muscles, trigeminal neuralgia, TMJ abnormality, and facial asymmetry.
Graham et al., 2021 [27]	United States	Cross‐sectional*	> 4 months	> 120 days	100	Mean 43.2 (11.3)	Non‐hospitalised	Anosmia and dysgeusia
Howe et al., 2021 [28]	United States	Cohort	1 year	365 days	115	≥ 18	Included all degrees of the disease	Anosmia and ageusia
Kaplan et al., 2021 [29]	Türkiye	Cross‐sectional*	> 12 Weeks	> 84 days	121	Mean 33.5 (22–59)	Included all degrees of the disease	Anosmia and ageusia
Klein et al., 2021 [30]	Israel	Cross‐sectional*	6 months	180 days	99	Mean 35 ± 12	Mild infection	Dysosmia and dysgeusia
Lemhöfer et al., 2021 [31]	Germany	Cross‐sectional	3 months	90 days	365	Mean 49.8 (± 16.9)	Mild and moderate infection	Dysosmia and dysgeusia
Lombardo et al., 2021 [32]	Italy	Cohort	> 3 months	> 90 days	303	Median 53 (42–63)	Included all degrees of the disease	Anosmia and dysgeusia
Messin et al., 2021 [33]	France	Cross‐sectional*	6 months	180 days	74	Mean 52.3 (± 18)	Included all degrees of the disease	Anosmia and ageusia
Peghin et al., 2021 [34]	Italy	Cohort	6 months	180 days	596	Mean 53 (± 15.8)	Included all degrees of the disease	Anosmia and dysgeusia
Romero‐Duarte et al., 2021 [35]	Spain	Cohort*	6 months	180 days	797	Mean 63.0 (± 14.4)	Hospitalised	Anosmia and dysgeusia
Schambeck et al., 2021 [36]	Germany	Cohort	100 days	100 days	44	≥ 18	Included all degrees of the disease	Anosmia and ageusia
Scherlinger et al., 2021 [37]	France	Cohort	102 days	102 days	30	Mean 40 (35–54)	Included all degrees of the disease	Anosmia and ageusia
Vaes et al., 2021 [38]	Belgium	Cohort	3 months	90 days	1005	Median 50.0 (39–56)	Hospitalised and Non‐hospitalised	Anosmia and ageusia
Akınci, T., 2022 [39]	Istanbul	Cohort*	3 months	90 days	85	Mean 47.5 (± 13.9)	Patients hospitalised for COVID‐19 and experiencing headaches	Anosmia and ageusia
Akova, I.; Gedikli, M.A., 2022 [40]	Türkiye	Cross‐sectional	12 Weeks	84 days	151	Mean 37.4 (± 11.2)	Included all degrees of the disease	Anosmia and ageusia
Alabsi et al., 2022 [41]	Saudi Arabia	Cross‐sectional	> 7 months	> 210 days	100	≥ 18	Included all degrees of the disease	Dysgeusia, dysosmia, anosmia and ageusia
Ali et al., 2022 [42]	United States	Cohort*	6–9 months	180–270 days	52	Mean 42.8 (± 11.5)	Except patients hospitalised for pneumonia or hypoxaemia	Anosmia and ageusia
Al‐Mahalawy et al., 2022 [43]	Egypt	Case series	12 Weeks	84 days	12	≥ 18	Included all degrees of the disease	Spontaneous osteoradionecrosis
AlRadini et al., 2022 [44]	Saudi Arabia	Cross‐sectional	12 Weeks	84 days	1000	≥ 18	Included all degrees of the disease	Anosmia and ageusia
Arjun et al., 2022 [45]	India	Cohort	6 months	180 days	371	≥ 18	Included all degrees of the disease	Dysgeusia, dysosmia/parosmia
Becker J et al., 2022 [46]	Germany	Case report	7 months	210 days	1	48	Non‐hospitalised	Anosmia and ageusia
Bellan M. et al., 2022 [47]	Italy	Cohort	> 12 weeks	90 days	324	≥ 18	Hospitalised	Ageusia, dysgeusia and anosmia
Boscolo‐Rizzo P. et al., 2022a [48]	Italy	Cross‐sectional	3 months	94–439 days	105	≥ 18	Included all degrees of the disease	Hypogeusia
Boscolo‐Rizzo P. et al., 2022b [49]	Italy	Cohort*	24 months	730 days	253	≥ 18	Non‐hospitalised	Anosmia and dysgeusia
Bussiere N. et al., 2022 [50]	Quebec	Cohort	11 months	330 days	366	Mean 44 (± 11.7)	Uninformed	Dysgeusia, dysosmia/parosmia
Caspersen et al., 2022 [51]	Norway	Cohort	11–12 months	330–360 days	774	> 18	Included all degrees of the disease	Anosmia and ageusia
Chaughtai S. et al., 2022 [52]	United States	Case report	6 months	180 days	1	30	Non‐hospitalised	Sensitivity and visually active tongue lesions
Chaumont et al., 2022 [53]	France	Cohort*	6 months	180 days	60	Mean 66 (55–73)	Included all degrees of the disease with neurological manifestation	Anosmia and ageusia
Chudzik M. et al., 2022 [54]	Poland	Cross‐sectional*	3 months	90 days	2218	≥ 18	Hospitalised and non‐hospitalised	Anosmia and ageusia
Damanti S. et al., 2022 [55]	Italy	Cohort	6 months	180 days	148	≥ 65	Hospitalised	Anosmia and ageusia
Desgranges F. et al., 2022 [6]	Switzerland	Cohort	3–10 months	90–300 days	418	Median 36 (29–47)	Non‐hospitalised	Anosmia and ageusia
Estrada‐Codecido J. et al., 2022 [56]	Canada	Cohort	3 months	90 days	466	Median 38 (27–57)	Hospitalised and non‐hospitalised	Anosmia and dysgeusia
Fang X. et al., 2022 [57]	China	Cohort	12 months	365 days	1233	≥ 60	Hospitalised	Dysgeusia and dysosmia/parosmia
Fanous J. et al., 2022 [58]	Canada	Case report	12 months	365 days	1	27	Critically ill patient but not hospitalised	Anosmia and ageusia
Fernández‐de‐las‐Peñas C. et al., 2022 [59]	Spain	Cohort	6 months	180 days	201	Median: 56.5 (± 21)	Hospitalised	Anosmia and ageusia
Fernández‐de‐las‐Peñas C. et al., 2022 [60]	Spain	Cross‐sectional	8 months	240 days	1969	Median: 61 (± 16)	Hospitalised	Anosmia and ageusia
Fernández‐de‐las‐Peñas et al., 2022 [61]	Spain	Cohort	5–10 months	150–300 days	1593	Mean 61 (± 16)	Included all degrees of the disease	Anosmia and ageusia
Fernández‐de‐las‐Peñas et al., 2022 [62]	Spain	Cross‐sectional*	6 months	180 days	614	> 18	Included all degrees of the disease	Anosmia and ageusia
Ferrucci R. et al., 2022 [63]	Italy	Cohort	5–12 months	150–360 days	129	≥ 18	Hospitalised	Dysgeusia and hyposmia
Fornazieri MA. et al., 2022 [64]	Brazil	Cohort	3 months	90 days	73	Median: 41.5 (± 13.8)	Included all degrees of the disease	Dysgeusia and dysosmia/parosmia
Förster et al., 2022 [65]	Germany	Cross‐sectional*	> 12 Weeks	> 84 days	1459	Mean 53 (37–62)	Included all degrees of the disease	Anosmia and ageusia
Fortunato et al., 2022 [66]	Italy	Cross‐sectional*	12 months	360 days	653	Mean 42.9 (± 17.9)	Mild and moderate infection in home quarantine	Anosmia and ageusia
Ghanem J. et al., 2022 [67]	France	Cohort	6 months	180 days	37	≥ 18	Hospitalised	Anosmia and ageusia
Grisanti, et al., 2022 [68]	Italy	Cross‐sectional*	3 months	90 days	3818	≥ 18	Hospitalised and non‐hospitalised	Anosmia and ageusia
Gudziol et al., 2022 [69]	Germany	Cohort	4 months	120 days	43	Median: 62 (± 14.7)	Hospitalised and non‐hospitalised	Dysosmia/parosmia and hypogeusia
Hintschich et al., 2022 [70]	Germany	Cross‐sectional	8 months	240 days	303	≥ 18	Hospitalised and non‐hospitalised	Hypogeusia and anosmia
Huang et al., 2022 [71]	China	Cohort	2 years	730 days	1192	Mean 57 (48–65)	Included all degrees of the disease	Anosmia, ageusia, and difficulty swallowing
Imoto et al., 2022 [72]	Japan	Cross‐sectional	12 months	360 days	285	Median 60 (46–76)	Hospitalised and non‐hospitalised	Anosmia and dysgeusia
Jabali et al., 2022 [73]	Saudi Arabia	Cross‐sectional	6 months	180 days	327	Mean 46.83 (± 16.5)	Included all degrees of the disease	Anosmia and ageusia
Kalak et al., 2022 [74]	Israel	Cohort	3–18 months	90–545 days	166	≥ 18	Included all degrees of the disease	Anosmia and ageusia
Kim et al., 2022 [75]	Korea	Cohort	12 months	365 days	170	≥ 18	Included all degrees of the disease	Anosmia and ageusia
Klopfenstein et al., 2022 [76]	France	Cross‐sectional*	9 months	270 days	214	Mean 48.8 (± 18.7)	Included all degrees of the disease	Dysgeusia e ageusia
Larsson S. et al., 2022 [77]	Sweden	Cohort	> 12 weeks	90 days	1584	≥ 18	Hospitalised and non‐hospitalised	Anosmia and ageusia
Lindahl A. et al., 2022 [78]	Finland	Cohort	6 months	180 days	101	≥ 18	Hospitalised	Anosmia and ageusia
Miyazato et al., 2022 [79]	Japan	Cross‐sectional	6 months	180 days	457	Median 47	Included all degrees of the disease	Dysosmia and dysgeusia
Nguyen et al., 2022 [80]	France	Cohort	6 months	180 days	605	≥ 18	Hospitalised and non‐hospitalised	Dysosmia/parosmia and dysgeusia
Olavegogeascoechea et al., 2022 [81]	Argentina	Cross‐sectional	> 12 weeks	90 days	1539	≥ 18	Hospitalised and non‐hospitalised	Dysosmia/parosmia and dysgeusia
Peluso et al., 2022 [82]	United States	Cohort	4 months	120 days	179	Median: 48 (37–57)	Hospitalised and non‐hospitalised	Anosmia and dysgeusia
Peters et al., 2022 [83]	Germany	Cross‐sectional	9 months	270 days	1406	≥ 18	Hospitalised and non‐hospitalised	Anosmia and ageusia
Rafałowicz et al., 2022 [84]	Poland	Case report	116–192 days	116–192 days	3	Mean 52	Mild and moderate infection	Unilateral sore‐like lesions with inflamed edges were found on the left side of the hard palate. Angioma‐type lesion on the right side of the palate. Extensive vascular‐type changes in the hard palate with spontaneous bleeding
Righi et al., 2022 [85]	Italy	Cohort	9 months	270 days	465	Mean 56 (45–66)	Hospitalised and non‐hospitalised	Dysgeusia and ageusia
Sadat Larijani et al., 2022 [86]	Iran	Cross‐sectional	> 24 weeks	168 days	254	≥ 18	Hospitalised and non‐hospitalised	Anosmia and ageusia
Sakurada et al., 2022 [87]	Japan	Cross‐sectional	3 months	90 days	65	Median 39 (25, 54)	Included all degrees of the disease	Dysgeusia
Santana et al., 2022 [88]	Brazil	Case series	6 months	180 days	15	Mean 46.1 (± 5.2)	Mild and moderate infection	Hypogeusia, anosmia, ageusia
Schambeck et al., 2022 [89]	Germany	Cohort	3 months	100 ‐ 244–721 days	44	≥ 18	Non‐hospitalised	Anosmia and ageusia
Seang et al., 2022 [90]	France	Cohort	6 months	180 days	39	≥ 18	Non‐severe outpatients	Anosmia and ageusia
Shafiee et al., 2022 [91]	Iran	Case series	4 months	120 days	3	≥ 18	Hospitalised and non‐hospitalised	Anosmia and ageusia
Shah et al., 2022 [92]	Nepal	Cohort	3 months	90 days	300	≥ 18	Included all degrees of the disease	Anosmia and ageusia
Subramanian et al., 2022 [14]	United Kingdon	Cohort	12 Weeks	84 days	486,149	Mean 43.8 (± 16.9)	Non‐hospitalised	Dysgeusia, anosmia, xerostomia, and mouth ulcer
Titze de Almeida et al., 2022 [93]	Brazil	Cohort	5 months	150 days	236	≥ 18	Non‐hospitalised	Dysgeusia and ageusia
Vaira et al., 2022 [94]	Italy and Belgium	Case‐control	12 months	360 days	170	Median 39.9 (± 10.5)	Hospitalised	Dysgeusia, anosmia and dysosmia/parosmia
van Elst et al., 2022 [95]	Netherlands	Cross‐sectional	6–10 months	180–300 days	157	≥ 18	Hospitalised and non‐hospitalised	Dysgeusia, dysosmia/parosmia and Xerostomia/Dry mouth
Wong‐Chew et al., 2022 [96]	Mexico	Cohort	3 months	90 days	928	≥ 18	Hospitalised	Anosmia and dysgeusia
AlBahrani S. et al., 2023 [97]	Saudi Arabia	Cross‐sectional	3–6 months	90–180 days	243	Mean 36.1 (± 7.6)	Included all degrees of the disease	Dysgeusia
Aldahleh H. et al., 2023 [98]	Jordan	Case series	> 12 weeks	90 days	366	≥ 18	Included all degrees of the disease	Dysgeusia, dysosmia/parosmia
Asakura et al., 2023 [99]	Japan	Case‐control	3 months	90 days	4328	≥ 18	Included all degrees of the disease	Anosmia and ageusia
Bertuccio P. et al., 2023 [100]	Italy	Cohort*	3 months	90 days	323	≥ 18	Non‐hospitalised	Ageusia, dysgeusia and anosmia
Boscolo‐Rizzo P. et al., 2023 [101]	Italy	Case‐control	12–24 months	360–720 days	93	Median 49 (37–56)	Non‐hospitalised	Anosmia and ageusia
Boscolo‐Rizzo P. et al., 2023 [102]	Italy	Cohort	6 months	180 days	294	≥ 18	Non‐hospitalised	Dysgeusia, dysosmia, anosmia and ageusia
Bougea A. et al., 2023 [103]	Greece	Cross‐sectional	6 months	180 days	38	Mean 61 (± 8.70)	Hospitalised and non‐hospitalised	Anosmia/hyposmia and dysgeusia
Brunvoll SH. et al., 2023 [104]	Norway	Cohort	3–15 months	90–450 days	1420	Mean 50 (± 13.6)	Hospitalised and non‐hospitalised	Anosmia and dysgeusia
Duwel V. et al., 2023 [105]	Aruba	Cohort	3‐6‐12–18 months	90‐180‐360–540 days	222	Median 58.1(± 12.8)	Hospitalised	Anosmia and ageusia
Elnadi H. et al., 2023 [106]	Cameroon, Egypt, Nigeria and Somalia	Cross‐sectional	3 months	90 days	713	≥ 18	Included all degrees of the disease	Anosmia and ageusia
Fernández‐de‐las‐Peñas C. et al., 2023 [107]	Spain	Cohort	18 months	540 days	1969	Median: 61 (± 16)	Hospitalised	Anosmia and ageusia
Feter N. et al., 2023 [108]	Brazil	Cohort	3–6 months	90–180 days	237	≥ 18	Uninformed	Anosmia and ageusia
Gottlieb et al., 2023 [109]	United States	Cross‐sectional*	6 months	180 days	5963	≥ 18	Hospitalised and non‐hospitalised	Anosmia and ageusia
Heeney et al., 2023 [110]	Ireland	Cohort	3 months	95 days	311	Median: 43 (31–53)	Hospitalised and non‐hospitalised	Anosmia and ageusia
Khanafer et al., 2023 [111]	France	Case‐control	12 months	365 days	178	63.4 (25–93)	Hospitalised	Anosmia and ageusia
Lapa J. et al., 2023 [112]	Brazil	Cohort	3–6 months	90–180 days	400	≥ 18	Hospitalised	Anosmia and ageusia
Nayani et al., 2023 [113]	Belgium	Cohort	3 months	90 days	3039	≥ 18	Hospitalised and non‐hospitalised	Ageusia, anosmia and difficulty/pain when swallowing or chewing
Nordvig et al., 2023 [114]	United States	Cohort	12 months	360 days	530	≥ 18	Hospitalised and non‐hospitalised	Anosmia and ageusia
Pagen et al., 2023 [115]	Netherlands	Cross‐sectional	3 months	90 days	2322	≥ 18	Hospitalised and non‐hospitalised	Dysosmia/parosmia and dysgeusia
Perez et al., 2023 [116]	United States	Cross‐sectional	6 months	180 days	600	Median 46.4 (± 14.0)	Hospitalised and non‐hospitalised	Anosmia and dysgeusia
Quinones‐Moya et al., 2023 [117]	Mexico	Cross‐sectional	3 months	90 days	64	≥ 18	Included all degrees of the disease	Dysosmia/parosmia and dysgeusia
Rass et al., 2023 [118]	Italy and Austria	Cohort	≥ 90 days	≥ 90 days	906	Austria: Median 43 (32–53); Italy: Median 45 (34–54)	Non‐hospitalised	Dysosmia and dysgeusia
Sari et al., 2023 [119]	Türkiye	Case‐control	3–6 months	90–180 days	40	≥ 18	Included all degrees of the disease	Periodontitis
Seller et al., 2023 [120]	Switzerland	Cohort	30 months	900 days	110	≥ 18	Included all degrees of the disease	Anosmia and ageusia
Silva et al., 2023 [121]	Brazil	Cohort	12 months	360 days	1371	Median 39.7 (± 11.7)	Hospitalised and non‐hospitalised	Anosmia and ageusia
Thi Khanh et al., 2023 [122]	Belgium	Cohort	3 months	90 days	3708	≥ 18	Hospitalised and non‐hospitalised	Anosmia and ageusia
van Zon et al., 2023 [123]	Netherlands	Cohort	3 months	90–150 days	624	Median: 55.8 (± 11.6)	Hospitalised and non‐hospitalised	Anosmia and ageusia
Wang et al., 2023 [124]	China	Cross‐sectional	24 months	360 days	1546	Median 57.93 (± 13.55)	Hospitalised	Anosmia and dysgeusia

*Classified by the authors of the systematic review, when not indicated in the article.

**Age values are presented as reported in the original articles. When only general information was available, we used descriptive labels (e.g., ≥ 18).

***Adapted by the authors of the systematic review.

Regarding the sex distribution of participants, the proportion of female participants ranged from 10% to 95.5%, with a median of 54.4% (Supporting Information S2). However, few studies provided sex‐disaggregated data for specific oral symptoms, which limited the possibility of performing subgroup analyses by sex.

Regarding the territory, most surveys were conducted in Europe (55.1%), followed by the Middle East (14.0%). The regions with the fewest studies were South America (6.5%), Africa (1.8%), and Caribbean (0.9%) (Table 1).

It was found that cohorts were the most frequent (54.2%), followed by cross‐sectional studies (32.7%). Reports and/or case series were the least frequent (13.0%). Regarding the minimum time used to define the case of post‐COVID‐19 condition, 12 weeks (32 studies; 29.9%) and 24 weeks (24 studies; 22.4%) stand out (Table 1).

Other outcomes related to oral manifestations were identified in a smaller number of studies, which did not allow for meta‐analysis. Among these manifestations, hyposalivation or dry mouth were mentioned in 4 studies [12, 13, 14, 95]. Tongue mucosal changes [13, 52], sensitivity in the trigeminal nerve [13, 50], and mouth ulcers or sore‐like lesions [14, 84] were reported in two studies each. Facial tremors, facial muscle weakness, facial asymmetry, and changes in the temporomandibular joint (TMJ) were mentioned in a single study [13] (Table 2).

**TABLE 2:** Number of studies evaluating different oral manifestations in patients with post COVID‐19 condition. Ponta Grossa‐Parana, Brazil: 2024.

Oral manifestation	Number of studies reporting this symptom
Taste alteration (ageusia, dysgeusia, hypogeusia)	104
Dry mouth (xerostomia)	4
Mouth ulcers or sore‐like lesions	2
Tongue mucosal changes	2
Difficulty swallowing (dysphagia)	2
Sensitivity in the trigeminal nerve	2
Weakness in the masticatory muscles	1
Facial tingling	1
Temporomandibular joint (TMJ) disorder	1
Facial asymmetry	1
Spontaneous osteoradionecrosis	1
Angioma‐like lesion	1
Lesions in hard palate	1
Periodontitis	1

Outcomes cited only one time in the studies included spontaneous osteoradionecrosis [43], severe periodontal disease [119], palate lesions [84], and angioma‐like lesion [84] (Table 2).

### 3.1 Meta‐Analysis

For the meta‐analysis, symptoms of ageusia, dysgeusia, and changes in taste, were considered, and all studies were quantified in meta‐analysis. The samples were separated into groups (represented by the letter G) when they presented divisions between variants of SARS‐CoV‐2 viruses, place of evaluation of symptoms, and patients hospitalised or not (Supporting Information S2).

**FIGURE 2:**
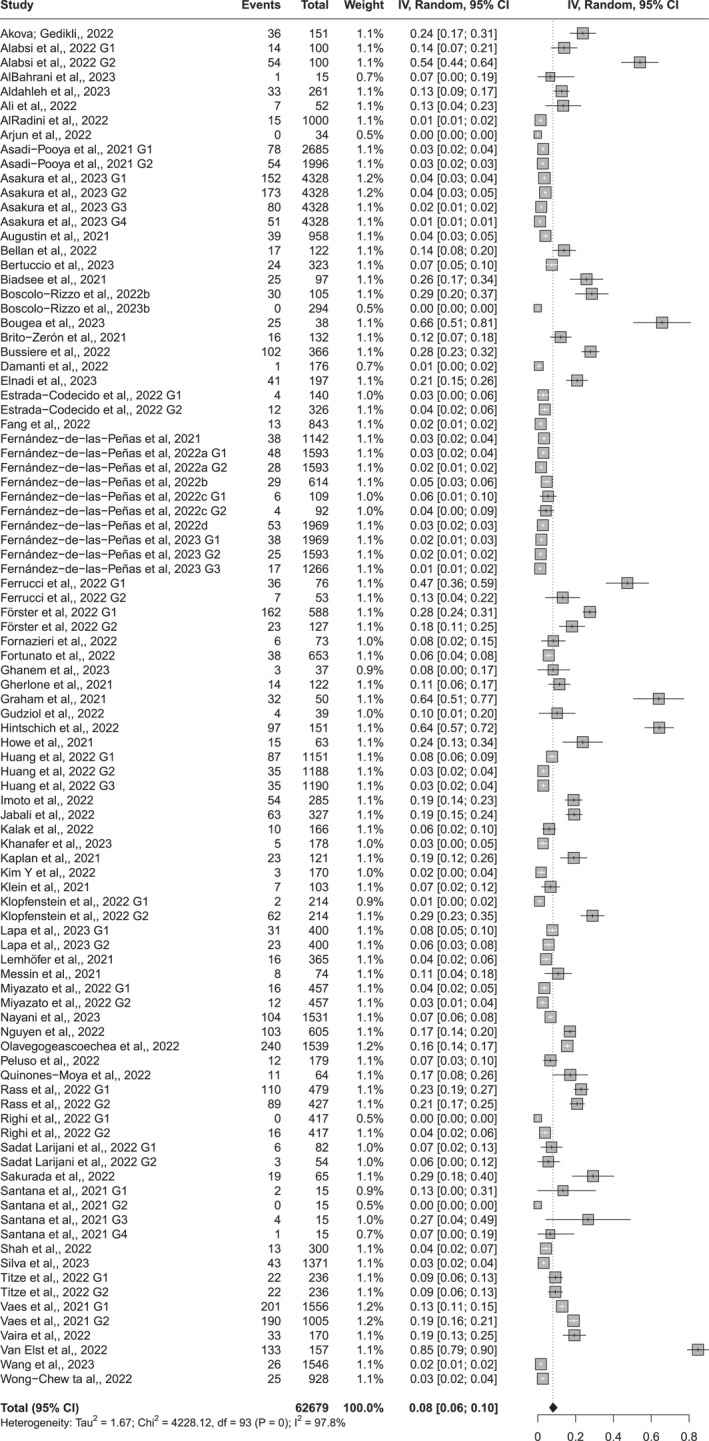
Forest Plot of the meta‐analysis of proportions of taste alteration symptoms, in adult individuals more than 12 weeks after the acute phase of COVID‐19.

Seventy‐two (72) studies were included in the meta‐analysis on changes in taste. Given the considerable clinical and methodological diversity among the included studies, as well as the high statistical heterogeneity (I^2^ = 97.8%), a random‐effects model was applied to estimate the pooled prevalence. An 8% prevalence (95% CI 6%–10%) of taste disorders was found, more than 12 weeks after the acute phase of COVID‐19 (Figure 2).

**FIGURE 3:**
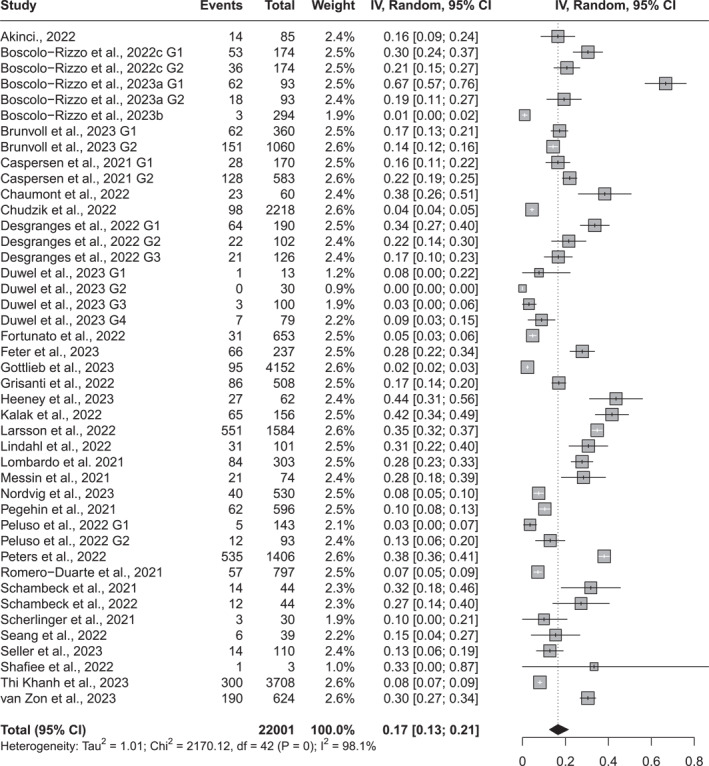
Forest Plot of the meta‐analysis of proportions of symptoms of altered taste and/or smell in adult individuals more than 12 weeks after the acute phase of COVID‐19.

In the meta‐analysis evaluating the prevalence of combined taste and smell alterations, substantial clinical and methodological heterogeneity was also anticipated across the included studies. Consistently, high statistical heterogeneity was observed (I^2^ = 98.1%). Therefore, a random‐effects model was used to estimate the pooled prevalence. More than 12 weeks after the acute phase of COVID‐19, 17% of individuals (95% CI: 13%–21%) reported some alteration in taste and/or smell as part of the post‐COVID‐19 condition (Figure 3).

Subgroup meta‐analyses were conducted for both outcomes. For taste alterations, the subgroups included risk of bias (high, moderate, and low), symptom type (ageusia, dysgeusia, and hypogeusia), number of study participants (up to 300 and more than 300), and time since acute infection (12–24 weeks, 24–48 weeks, and more than 48 weeks) (Supporting Information S4).

For the outcome of combined taste and/or smell alterations, subgroup analyses considered risk of bias (high, moderate, and low), number of participants (up to 500 and more than 500), and time since infection (12–24 weeks, 24–48 weeks, and more than 48 weeks) (Supporting Information S4).

Meta‐regression analyses were conducted to explore potential sources of heterogeneity. For taste alterations, the model explained 38.2% of the variance (*R* ^2^ = 38.16%; *p* < 0.001). A significantly lower prevalence was observed in studies assessing symptoms at 24–48 weeks (95% CI: −1.51 to −0.01; *p* = 0.048) and after more than 48 weeks (95% CI: −2.18 to −0.0026; *p* = 0.045), compared to studies with shorter follow‐up. Studies with more than 300 participants also reported lower prevalence (95% CI: −1.20 to −0.17; *p* = 0.009). Conversely, studies focussing on hypogeusia showed significantly higher prevalence (95% CI: 0.45 to 2.85; *p* = 0.007).

For the combined outcome of taste and smell alterations, residual heterogeneity remained high (I^2^ = 98.07%), and none of the tested moderators including risk of bias, sample size, or symptom duration, were statistically significant. The model did not explain any of the observed heterogeneity (*R* ^2^ = 0.00%; *p* = 0.608).

Regarding methodological quality, 40 studies (37.4%) were classified as having low risk of bias, 53 (49.5%) as moderate risk, and 14 (13.1%) as high risk (Supporting Information S2). The certainty of the evidence was assessed using the GRADE approach and was rated as moderate for both outcomes: taste alteration and combined taste and smell alteration (Supporting Information S3).

## 4 Discussion

The main findings of this systematic review underscore the high frequency of studies reporting taste and/or smell alterations, as well as the high prevalence of these symptoms as part of the post‐COVID‐19 condition. Conversely, there was a low frequency of studies addressing other oral manifestations following the acute phase of infection.

Taste alterations were the most prevalent oral symptoms during the acute phase of COVID‐19, affecting approximately 45% of patients [15, 125]. This manifestation persists in the post‐COVID‐19 condition, with over 8% of individuals experiencing some form of taste dysfunction three months or more after the acute phase, corroborating the 10.6% prevalence of taste alterations reported in another review [126].

Our findings also reveal a co‐occurrence between taste and smell dysfunctions. Taste alterations are often secondary to olfactory impairment [127]. The persistence of these symptoms may be associated with mechanisms such as excessive inflammatory cytokine production, neuroinflammation, and hypoxia caused by viral infection [125, 126, 128]. However, current evidence remains inconclusive, reinforcing the need for further research on this topic.

Persistent taste alterations can result in irritability, sadness, relationship difficulties, and other quality‐of‐life impairments. Recognising these outcomes highlights the essential role of dental professionals in supporting comprehensive health and patient recovery.

Xerostomia was found to have a high frequency in individual studies. If left untreated, it can cause significant discomfort. This review also identified other post‐COVID manifestations, including hyposalivation, osteoradionecrosis, facial paraesthesia, dysphagia, and oral ulcers, among others. Even though these symptoms appear at lower frequencies, patients have reported them in dental clinics, requiring appropriate treatment. Although not emergency conditions, these symptoms can have a substantial impact on quality of life and may demand complex rehabilitation. Dentists, as well as other healthcare professionals, should consider including questions about acute COVID‐19 and post‐COVID‐19 conditions in the clinical anamnesis.

The prognosis of post‐COVID‐19 conditions remains unclear and is likely influenced by the severity of clinical symptoms, underlying comorbidities, and individual treatment responses [129]. In a 2‐year follow‐up study after COVID‐19 infection, recovery of smell and taste was found to be most significant within the first 100 days post‐acute phase, with little or no improvement thereafter [36]. This suggests that the post‐COVID‐19 condition may become established after this period, and therapeutic interventions before the chronic phase could be more effective.

Ongoing training and continuing education for oral health professionals are essential. Training courses should address diagnostic methods, treatment, and follow‐up for post‐COVID‐19 conditions. Given that post‐COVID‐19 involves a wide range of potential multisystemic manifestations, all healthcare professionals should be prepared for appropriate oral health diagnosis and timely treatment or referral. Interprofessional collaboration aims to improve care delivery, reduce healthcare system burden, and avoid resource exhaustion, which had already been under significant strain during the pandemic [129].

More than 50% of the studies included in this review were cohort studies, suggesting a research interest in understanding the temporal role of post‐COVID‐19 oral manifestations and providing methodological potential for informing symptom and condition prognosis. Longer follow‐up periods may offer more information on symptom duration and disease outcomes but are subject to recall or measurement bias [36]. Moreover, self‐reported symptoms may not always be attributable to the post‐COVID‐19 condition. Future studies should include interventional designs, clinical monitoring, and laboratory data, and when possible, follow healthy cohorts to test causal hypotheses about oral manifestations in the post‐COVID‐19 condition. Multidisciplinary research teams are necessary to broadly investigate the subject, enabling diagnosis and therapeutic options in post‐COVID‐19 clinical care.

The length of follow‐up after the acute phase of the disease remains a controversial element in defining post‐COVID‐19 conditions [7, 10, 11]. This inconsistency may act as a methodological confounder in studies on the topic, and a global standardisation for disease definition is recommended. The scarcity of studies in Latin America and other regions with fewer research resources highlights the challenges posed by limited funding and difficulties in publishing in high‐impact journals [130]. During the pandemic, many dental surgeons likely prioritised clinical care over research due to socioeconomic pressures and activity disruptions [131, 132]. A 2020 review on taste and smell disorders included only studies from North America, Europe, and Asia [133], and this geographic imbalance persists in the current review.

Although some symptoms are common worldwide, the prevalence of acute COVID‐19 symptoms has varied by region; for instance, dysgeusia affected 16.2% in East Asia versus 50.3% in Western countries [134]. These differences may influence how post‐COVID‐19 conditions are measured and addressed, reinforcing the need for sensitive local surveillance systems. The lack of equitable epidemiological mapping of post‐COVID‐19 manifestations, including oral symptoms, hampers accurate global diagnosis and the development of therapeutic protocols. Despite vaccination advances and declining acute infection rates, an estimated 76 million people continue to suffer from prolonged symptoms [135, 136]. Addressing this public health challenge requires evidence‐based care strategies supported by ongoing research [137].

Post‐COVID‐19 conditions have received increasing attention in recent years, with more than half of the studies in this review published recently. Symptomatology may vary by viral variant, for example, Omicron is associated with milder symptoms [102], while Delta shows different patterns [61], as well as by vaccination period [61]. Understanding the variation across pandemic phases is essential for characterising viral behaviour and the variability of long‐term responses among individuals [138].

This study limited its meta‐analysis approach to taste alterations and the proportion of taste dysfunction associated with smell dysfunction. This indicates that these are the primary post‐COVID‐19 oral symptoms identified in the current literature, aligned with the definition of the post‐COVID‐19 condition. However, the study could not determine the extent of morbidity from other oral manifestations or the clinical service demand for their management, as they have received little research attention to date. Furthermore, there is a lack of diagnostic data and clinical studies providing therapeutic solutions and accurate diagnostic methods for oral manifestations of the post‐COVID‐19 condition.

Despite incorporating important moderators such as sample size, risk of bias, symptom duration, and type of dysfunction, a high degree of heterogeneity persisted in both meta‐analyses, indicating considerable variability across studies. In the analysis of taste dysfunction, 38.16% of heterogeneity was explained by the included moderators. Symptom duration and dysfunction type were significant factors, with longer follow‐up periods associated with lower symptom prevalence, suggesting spontaneous resolution over time. Additionally, hypogeusia was more strongly associated with symptom persistence compared to dysgeusia, indicating possible differences in underlying pathophysiology. Studies with smaller sample sizes (< 300 participants) reported higher symptom proportions, suggesting potential publication bias or greater statistical variability in smaller samples (Supporting Information S4).

In contrast, in the combined analysis of taste and smell alterations, none of the tested moderators successfully explained the heterogeneity, leaving most of the variability unaccounted for. This suggests that unmeasured factors—such as methodological differences, variations in diagnostic criteria, population characteristics, and geographic factors—may be influencing the results. As with isolated taste alterations, symptom prevalence tended to decline over time, which may reflect natural recovery, differences in follow‐up duration, or attrition bias. The persistent unexplained heterogeneity in the combined analysis highlights the complexity of synthesising prevalence data from diverse study designs and populations (Supporting Information S4).

Regarding the inclusion criteria, the decision to include only studies with participants who tested positive for COVID‐19 stems from the need to confirm COVID‐19 during the acute phase to establish a causal link with the post‐COVID‐19 condition (chronic manifestations). Excluding cases not confirmed through clinical‐epidemiological criteria increases the specificity of the COVID‐19 surveillance system, allowing stronger attribution of oral health findings to the initial COVID‐19 infection. Nonetheless, this does not prevent clinicians from using the results of this systematic review to assess individuals with suspected post‐COVID‐19 condition, even in the absence of prior confirmatory testing.

## 5 Conclusion

Taste alterations were the most frequently reported oral manifestation in post‐COVID‐19 condition, with a pooled prevalence of 8%, and combined taste and smell alterations showed a prevalence of 17%. These findings underscore the need for clinical recognition and appropriate management by oral health professionals, as they represent the most persistent and impactful symptoms among affected individuals. Other oral manifestations, such as hyposalivation, mouth ulcers, and facial tingling, were reported infrequently across studies. The limited mention of these conditions highlights the need for further research to clarify their prevalence, underlying mechanisms, and clinical implications in post‐COVID‐19 patients.

## Author Contributions

**Letícia Simeoni Avais:** Conceptualisation, literature review, research design, data collection and analysis, results interpretation, manuscript writing, and formatting. **Elis Carolina Pacheco:** Literature review, data collection, results interpretation, and manuscript writing. **Luisa Pereira de Oliveira Zanetti Gomes:** Data collection, and results interpretation. **Márcia Helena Baldani:** Study conceptualisation and final review of the manuscript. **Camila Marinelli Martins:** Research design, results interpretation, manuscript writing, and final proofreading. **Eliseu Alves Waldman:** Study conceptualisation and final review of the manuscript. **Pollyanna Kássia de Oliveira Borges:** Conceptualisation, research design, data collection, results interpretation, manuscript writing, and formatting. **Tomoko Y. Steen:** Critical review and revision of the manuscript contribution to the final discussion and overall scientific clarity. **Jean Paul J. Gonzalez:** Critical review and revision of the manuscript contribution to the contextualisation of findings from a global health perspective. All authors have reviewed and approved the final version of the manuscript and accept full responsibility for the content and any ethical concerns related to the research.

## Supporting information

Supporting Information S1

Supporting Information S2

Supporting Information S3

Supporting Information S4

## Data Availability

The data that support the findings of this study are available from the corresponding author, Letícia Simeoni Avais, upon reasonable request.
