# Supplementary material for: Oral Manifestations in the Post COVID‐19 Condition: A Systematic Review With Meta‐Analysis
Source: Rev Med Virol. 2025 Jul 15;35(4):e70057. doi: 10.1002/rmv.70057 (PMC12262108; doi:10.1002/rmv.70057)
Supplement: Supplementary file 1 — Supporting Information S1 [file RMV-35-e70057-s001.docx]

Supplemental Material 01.

*Search strategy in the databases*.

| **PUBMED/ MEDLINE** | | |
| --- | --- | --- |
| Search 1 |  | Search 2 |
| (((((((((((((((long covid) OR (persistent COVID-19 conditions) OR (after covid)) OR (long haul covid)) OR (post acute covid)) OR (post acute covid syndrome)) OR (persistent covid)) OR (long-COVID)) OR (long-haul COVID))) OR (post-acute COVID syndrome)) OR (persistent COVID-19)) OR (post-acute COVID19 syndrome)) OR (long hauler COVID)) OR (post-acute sequelae of SARS-CoV-2 infection)) OR (chronic COVID syndrome)) | **AND** | (((((((((((((Mouth) OR (Oral Diagnosis)) OR (Oral Manifestations)) OR (Oral Pathology)) OR (Xerostomia)) OR (Burning Mouth Syndrome)) OR (Oral Medicine)) OR (Mouth Mucosa)) OR (Salivary)) OR (dry mouth)) OR (Ageusia)) OR (Dysgeusia)) OR (Gland)) |

| **EMBASE** | | |
| --- | --- | --- |
| Search 1 |  | Search 2 |
| (long AND ('covid'/exp OR covid) OR 'persistent covid-19 conditions' OR (persistent AND ('covid 19'/exp OR 'covid 19') AND conditions) OR 'after covid' OR (after AND ('covid'/exp OR covid)) OR 'long haul covid'/exp OR 'long haul covid' OR (long AND haul AND ('covid'/exp OR covid)) OR 'post acute covid' OR (post AND acute AND ('covid'/exp OR covid)) OR 'post acute covid syndrome'/exp OR 'post acute covid syndrome' OR (post AND acute AND ('covid'/exp OR covid) AND ('syndrome'/exp OR syndrome)) OR 'persistent covid' OR (persistent AND ('covid'/exp OR covid)) OR 'long covid'/exp OR 'long covid' OR 'long-haul covid'/exp OR 'long-haul covid' OR ('long haul' AND ('covid'/exp OR covid)) OR 'post-acute covid syndrome'/exp OR 'post-acute covid syndrome' OR ('post acute' AND ('covid'/exp OR covid) AND ('syndrome'/exp OR syndrome)) OR 'persistent covid-19' OR (persistent AND ('covid 19'/exp OR 'covid 19')) OR 'post-acute covid19 syndrome' OR ('post acute' AND ('covid19'/exp OR covid19) AND ('syndrome'/exp OR syndrome)) OR 'long hauler covid'/exp OR 'long hauler covid' OR (long AND hauler AND ('covid'/exp OR covid)) OR 'post-acute sequelae of sars-cov-2 infection'/exp OR 'post-acute sequelae of sars-cov-2 infection' OR ('post acute' AND sequelae AND of AND ('sars cov 2'/exp OR 'sars cov 2') AND ('infection'/exp OR infection)) OR 'chronic covid syndrome'/exp OR 'chronic covid syndrome' OR (chronic AND ('covid'/exp OR covid) AND ('syndrome'/exp OR syndrome))) | **AND** | ('mouth'/exp OR mouth OR 'oral diagnosis'/exp OR 'oral diagnosis' OR (oral AND ('diagnosis'/exp OR diagnosis)) OR 'oral manifestations'/exp OR 'oral manifestations' OR (oral AND manifestations) OR 'oral pathology'/exp OR 'oral pathology' OR (oral AND ('pathology'/exp OR pathology)) OR 'xerostomia'/exp OR xerostomia OR 'burning mouth syndrome'/exp OR 'burning mouth syndrome' OR (('burning'/exp OR burning) AND ('mouth'/exp OR mouth) AND ('syndrome'/exp OR syndrome)) OR 'oral medicine'/exp OR 'oral medicine' OR (oral AND ('medicine'/exp OR medicine)) OR 'mouth mucosa'/exp OR 'mouth mucosa' OR (('mouth'/exp OR mouth) AND ('mucosa'/exp OR mucosa)) OR salivary OR 'dry mouth'/exp OR 'dry mouth' OR (dry AND ('mouth'/exp OR mouth)) OR 'ageusia'/exp OR ageusia OR 'dysgeusia'/exp OR dysgeusia OR 'gland'/exp OR gland) |

| **Web of Science** | | |
| --- | --- | --- |
| Search 1 |  | Search 2 |
| (((((((((((((((long covid) OR (persistent COVID-19 conditions) OR (after covid)) OR (long haul covid)) OR (post acute covid)) OR (post acute covid syndrome)) OR (persistent covid)) OR (long-COVID)) OR (long-haul COVID))) OR (post-acute COVID syndrome)) OR (persistent COVID-19)) OR (post-acute COVID19 syndrome)) OR (long hauser COVID)) OR (post-acute sequelae of SARS-CoV-2 infection)) OR (chronic COVID syndrome)) (All Fields) | **AND** | (((((((((((((Mouth) OR (Oral Diagnosis)) OR (Oral Manifestations)) OR (Oral Pathology)) OR (Xerostomia)) OR (Burning Mouth Syndrome)) OR (Oral Medicine)) OR (Mouth Mucosa)) OR (Salivary)) OR (dry mouth)) OR (Ageusia)) OR (Dysgeusia)) OR (Gland)) (All Fields) |

| **LILACS** | | | | |
| --- | --- | --- | --- | --- |
| Search 1 |  | Search 2 |  |  |
| (((((((((((((((long covid) OR (persistent covid-19 conditions) OR (after covid)) OR (long haul covid)) OR (post acute covid)) OR (post acute covid syndrome)) OR (persistent covid)) OR (long-covid)) OR (long-haul covid))) OR (post-acute covid syndrome)) OR (persistent covid-19)) OR (post-acute covid19 syndrome)) OR (long hauler covid)) OR (post-acute sequelae of sars-cov-2 infection)) OR (chronic covid syndrome)) | **AND** | (((((((((((((mouth) OR (oral diagnosis)) OR (oral manifestations)) OR (oral pathology)) OR (xerostomia)) OR (burning mouth syndrome)) OR (oral medicine)) OR (mouth mucosa)) OR (salivary)) OR (dry mouth)) OR (ageusia)) OR (dysgeusia)) OR (gland)) | **AND** | instance: "lilacsplus" |

| **COCHRANE** | | | | |
| --- | --- | --- | --- | --- |
| Search 1 |  | Search 2 |  |  |
| (((((((((((((((long covid) OR (persistent COVID-19 conditions) OR (after covid)) OR (long haul covid)) OR (post acute covid)) OR (post acute covid syndrome)) OR (persistent covid)) OR (long-COVID)) OR (long-haul COVID))) OR (post-acute COVID syndrome)) OR (persistent COVID-19)) OR (post-acute COVID19 syndrome)) OR (long hauler COVID)) OR (post-acute sequelae of SARS-CoV-2 infection)) OR (chronic COVID syndrome)) | **AND** | (((((((((((((Mouth) OR (Oral Diagnosis)) OR (Oral Manifestations)) OR (Oral Pathology)) OR (Xerostomia)) OR (Burning Mouth Syndrome)) OR (Oral Medicine)) OR (Mouth Mucosa)) OR (Salivary)) OR (dry mouth)) OR (Ageusia)) OR (Dysgeusia)) OR (Gland)) | **IN** | All Text |

| **SCOPUS** | | |
| --- | --- | --- |
| Search 1 |  | Search 2 |
| ( ALL ( ( ( ( ( ( ( ( ( ( ( ( ( ( ( ( long AND covid ) OR ( persistent AND covid-19 AND conditions ) OR ( after AND covid ) ) OR ( long AND haul AND covid ) ) OR ( post AND acute AND covid ) ) OR ( post AND acute AND covid AND syndrome ) ) OR ( persistent AND covid ) ) OR ( long-covid ) ) OR ( long-haul AND covid ) ) ) OR ( post-acute AND covid AND syndrome ) ) OR ( persistent AND covid-19 ) ) OR ( post-acute AND covid19 AND syndrome ) ) OR ( long AND hauler AND covid ) ) OR ( post-acute AND sequelae AND of AND sars-cov-2 AND infection ) ) OR ( chronic AND covid AND syndrome ) ) ) | **AND** | (ALL ( ( ( ( ( ( ( ( ( ( ( ( ( ( mouth ) OR ( oral AND diagnosis ) ) OR ( oral AND manifestations ) ) OR ( oral AND pathology ) ) OR ( xerostomia ) ) OR ( burning AND mouth AND syndrome ) ) OR ( oral AND medicine ) ) OR ( mouth AND mucosa ) ) OR ( salivary ) ) OR ( dry AND mouth ) ) OR ( ageusia ) ) OR ( dysgeusia ) ) OR ( gland ) ) ) ) |
