# Supplementary material for: Oral Manifestations in the Post COVID‐19 Condition: A Systematic Review With Meta‐Analysis
Source: Rev Med Virol. 2025 Jul 15;35(4):e70057. doi: 10.1002/rmv.70057 (PMC12262108; doi:10.1002/rmv.70057)
Supplement: Supplementary file 3 — Supporting Information S3 [file RMV-35-e70057-s003.pdf]

Question: Course of oral health manifestations in Long COVID over more than 3 mothes
Setting: Long COVID-19 presents as symptoms, new or persistent for a period greater than 3 months after the acute phase of COVID-19 disease. The objective of the systematic review is to evaluate the frequencies of oral manifestations reported in the literature that are characterized as manifestations of long COVID.
Bibliography: World Health Organization. A clinical case definition of post COVID-19 condition by a Delphi consensus. World Health Organization. Published October 6, 2021. Accessed October 2, 2023. https://www.who.int/publications/i/item/WHO-2019-nCoV-Post_COVID-19_condition-Clinical_case_definition-2021.1
Author(s): Avais, L S; Pacheco, E C; Gomes, L P O Z; Martins, C M; Baldani, M H; Borges, P K O.

№ of studies	Certainty assessment						Effect			Certainty	Importance
	Study design	Risk of bias	Inconsistency	Indirectness	Imprecision	Other considerations	№ of events	№ of individuals	Rate (95% CI)		
Loss or change in taste (follow-up: range 3 months to 2 years; assessed with: number of cases)											
72	non-randomised studies	not serious[a]	serious[b]	not serious	not serious	none	4533	449738	event rate 0,08 per (0.06 to 0.1)	⨁⨁⨁◯ Moderate[a,b]	IMPORTANT
Taste and Smell Alteration (follow-up: range 3 months to 2 years; assessed with: number of cases)											
34	non-randomised studies	not serious[a]	serious[b]	not serious	not serious	none	3102	22001	event rate 0,17% (0.13 to 0.21)	⨁⨁⨁◯ Moderate[a,b]	IMPORTANT

Explanations

a. The risk of bias assessment indicated the presence of studies with high, moderate, and low risk. However, studies with a high risk of bias have a relatively small weight in the overall analysis, reducing their influence on the combined effect estimate. Therefore, it was not necessary to downgrade the certainty of the evidence due to this limitation
b. The inconsistency across studies was high, as indicated by persistently elevated I^2 values in all analyses (Supplementary Material 4). Despite conducting subgroup analyses and meta-regression, the heterogeneity remained largely unexplained, with no significant moderators identified. Moreover, the overlap of confidence intervals across subgroups suggests that variability in effects was not fully accounted for by study characteristics such as sample size or risk of bias. Given the methodological diversity introduced by the pandemic context—where studies were conducted under varying conditions and employed different diagnostic approaches—the overall inconsistency was considered serious.
