# Supplementary material for: Oral Manifestations in the Post COVID‐19 Condition: A Systematic Review With Meta‐Analysis
Source: Rev Med Virol. 2025 Jul 15;35(4):e70057. doi: 10.1002/rmv.70057 (PMC12262108; doi:10.1002/rmv.70057)
Supplement: Supplementary file 4 — Supporting Information S4 [file RMV-35-e70057-s002.pdf]

Meta-Regressions

TASTE ALTERATIONS

```
Mixed-Effects Model (k = 94; tau^2 estimator: REML)

tau^2 (estimated amount of residual heterogeneity):     1.0358 (SE = 0.1753)
tau (square root of estimated tau^2 value):             1.0177
I^2 (residual heterogeneity / unaccounted variability): 96.88%
H^2 (unaccounted variability / sampling variability):   32.10
R^2 (amount of heterogeneity accounted for):            38.16%

Test for Residual Heterogeneity:
QE(df = 86) = 2109.9787, p-val < .0001

Test of Moderators (coefficients 2:8):
QM(df = 7) = 51.2433, p-val < .0001

Model Results:

                                          estimate      se     zval    pval    ci.lb    ci.ub
intrcpt                                    -1.7357  0.3531  -4.9159  <.0001  -2.4277  -1.0437  ***
dadosTaste$BiasLow                         -0.5682  0.3996  -1.4218  0.1551  -1.3514   0.2150
dadosTaste$BiasModerate                    -0.5311  0.3621  -1.4668  0.1424  -1.2409   0.1786
dadosTaste$Time24 to 48 weeks              -0.7576  0.3835  -1.9755  0.0482  -1.5093  -0.0060    *
dadosTaste$Timemore than 48 weeks          -1.0924  0.5560  -1.9647  0.0495  -2.1822  -0.0026    *
dadosTaste$NsampleMore than 300 patients   -0.6832  0.2617  -2.6111  0.0090  -1.1961  -0.1704   **
dadosTaste$DisfunctionTypeDysgeusia         0.4855  0.2466   1.9688  0.0490   0.0022   0.9689    *
dadosTaste$DisfunctionTypeHypogeusia        1.6501  0.6139   2.6880  0.0072   0.4469   2.8532   **

---
Signif. codes:  0 ‘***’ 0.001 ‘**’ 0.01 ‘*’ 0.05 ‘.’ 0.1 ‘ ’ 1
```

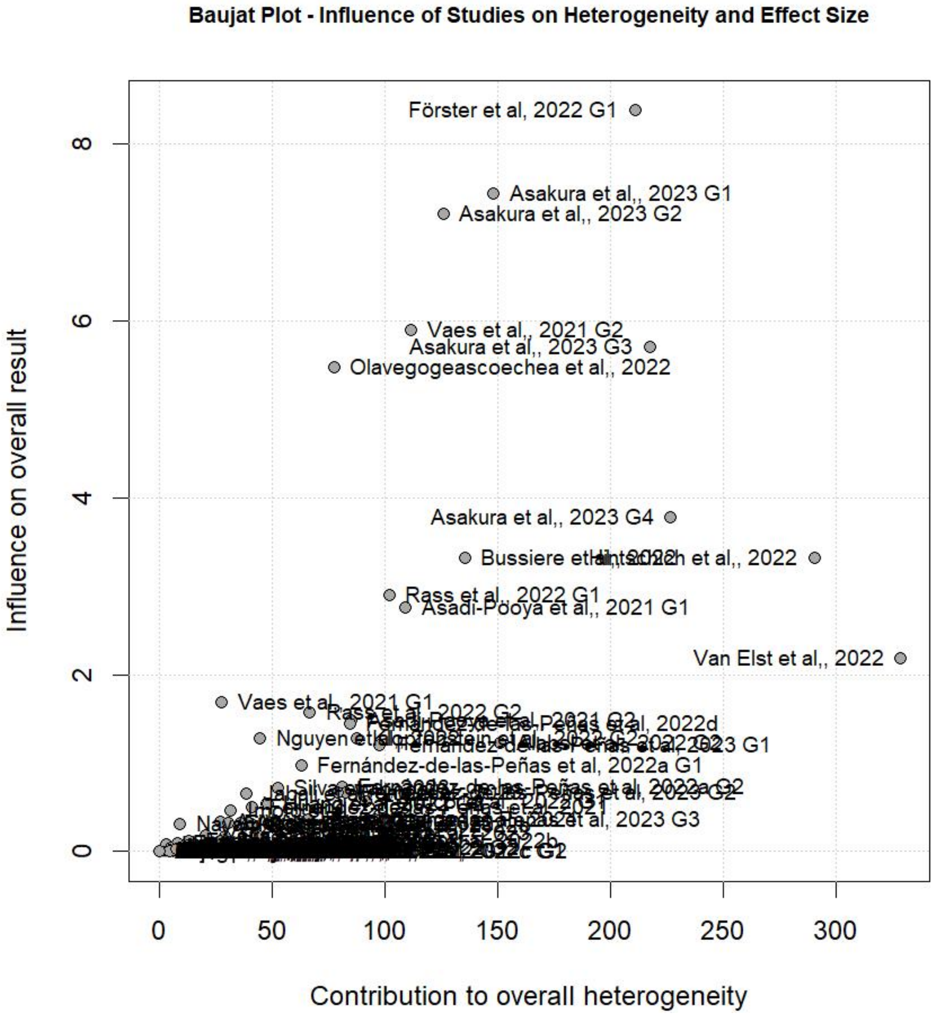

TASTE AND SMELL ALTERATIONS

```
Mixed-Effects Model (k = 43; tau^2 estimator: REML)

tau^2 (estimated amount of residual heterogeneity):     1.0386 (SE = 0.2646)
tau (square root of estimated tau^2 value):             1.0191
I^2 (residual heterogeneity / unaccounted variability): 98.07%
H^2 (unaccounted variability / sampling variability):   51.94
R^2 (amount of heterogeneity accounted for):            0.00%

Test for Residual Heterogeneity:
QE(df = 37) = 1949.7295, p-val < .0001

Test of Moderators (coefficients 2:6):
QM(df = 5) = 3.6006, p-val = 0.6082

Model Results:

                                             estimate      se     zval    pval    ci.lb   ci.ub
intrcpt                                       -1.0569  0.7645  -1.3825  0.1668  -2.5554  0.4415
dadosTasteSmell$BiasLow                       -0.7066  0.8824  -0.8008  0.4233  -2.4361  1.0229
dadosTasteSmell$BiasModerate                  -0.3981  0.8073  -0.4932  0.6219  -1.9803  1.1840
dadosTasteSmell$Time24 to 48 weeks             0.2446  0.5521   0.4430  0.6578  -0.8376  1.3268
dadosTasteSmell$TimeMore than 48 weeks         0.0973  0.6093   0.1597  0.8731  -1.0968  1.2914
dadosTasteSmell$NsampleMore than 500 patients -0.4517  0.3900  -1.1584  0.2467  -1.2161  0.3126

---
Signif. codes:  0 ‘***’ 0.001 ‘**’ 0.01 ‘*’ 0.05 ‘.’ 0.1 ‘ ’ 1
```

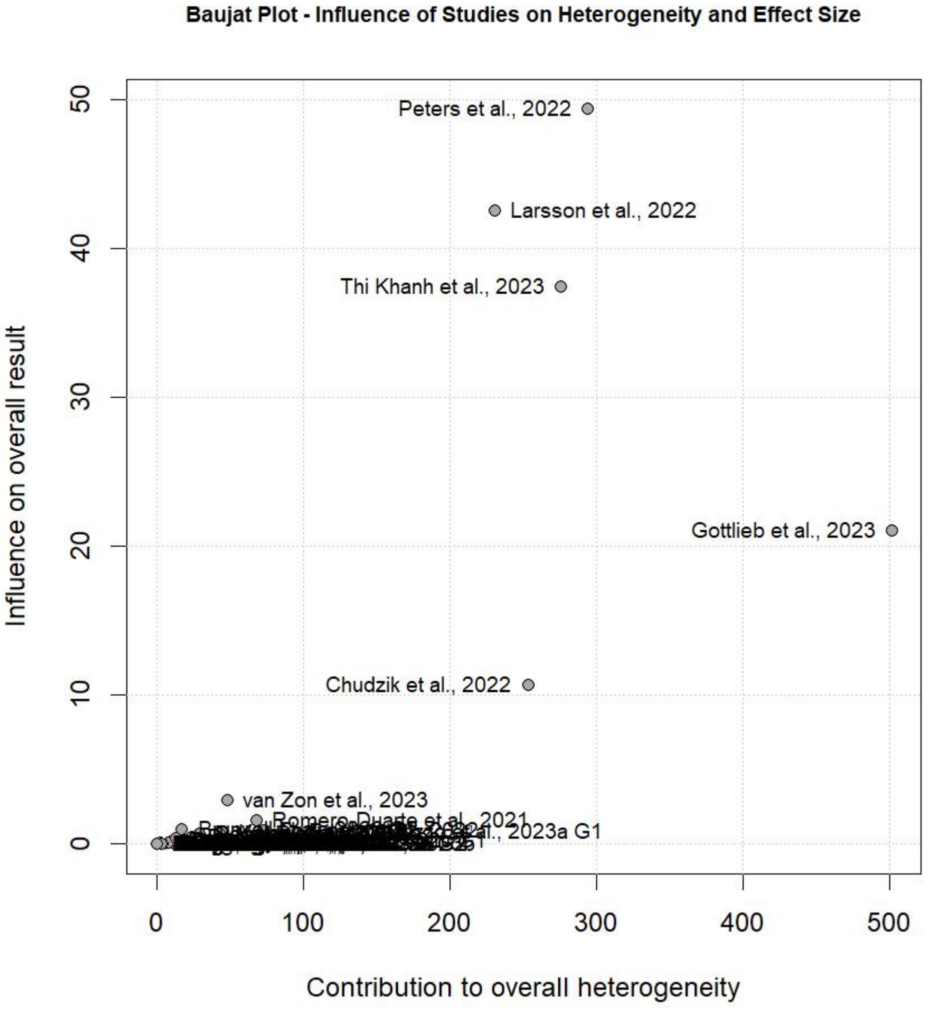

Study or Subgroup	Events	Total	Weight	IV, Random, 95% CI
**Bias = Low**				
Akova; Gedikli,, 2022	36	151	1.1%	0.24 [0.17; 0.31]
Ali et al,, 2022	7	52	1.1%	0.13 [0.04; 0.23]
AlRadini et al,, 2022	15	1000	1.1%	0.01 [0.01; 0.02]
Arjun et al,, 2022	0	34	0.5%	0.00 [0.00; 0.00]
Asadi−Pooya et al,, 2021 G1	78	2685	1.1%	0.03 [0.02; 0.04]
Asadi−Pooya et al,, 2021 G2	54	1996	1.1%	0.03 [0.02; 0.03]
Augustin et al,, 2021	39	958	1.1%	0.04 [0.03; 0.05]
Boscolo−Rizzo et al,, 2022b	30	105	1.1%	0.29 [0.20; 0.37]
Boscolo−Rizzo et al,, 2023b	0	294	0.5%	0.00 [0.00; 0.00]
Elnadi et al,, 2023	41	197	1.1%	0.21 [0.15; 0.26]
Fernández−de−las−Peñas et al, 2022a G1	48	1593	1.1%	0.03 [0.02; 0.04]
Fernández−de−las−Peñas et al, 2022a G2	28	1593	1.1%	0.02 [0.01; 0.02]
Fernández−de−las−Peñas et al, 2022b	29	614	1.1%	0.05 [0.03; 0.06]
Fernández−de−las−Peñas et al, 2022d	53	1969	1.1%	0.03 [0.02; 0.03]
Fernández−de−las−Peñas et al, 2023 G1	38	1969	1.1%	0.02 [0.01; 0.03]
Fernández−de−las−Peñas et al, 2023 G2	25	1593	1.1%	0.02 [0.01; 0.02]
Fernández−de−las−Peñas et al, 2023 G3	17	1266	1.1%	0.01 [0.01; 0.02]
Förster et al, 2022 G1	162	588	1.1%	0.28 [0.24; 0.31]
Förster et al, 2022 G2	23	127	1.1%	0.18 [0.11; 0.25]
Fornazieri et al,, 2022	6	73	1.0%	0.08 [0.02; 0.15]
Fortunato et al,, 2022	38	653	1.1%	0.06 [0.04; 0.08]
Graham et al,, 2021	32	50	1.1%	0.64 [0.51; 0.77]
Gudziol et al,, 2022	4	39	1.0%	0.10 [0.01; 0.20]
Jabali et al,, 2022	63	327	1.1%	0.19 [0.15; 0.24]
Lapa et al,, 2023 G1	31	400	1.1%	0.08 [0.05; 0.10]
Lapa et al,, 2023 G2	23	400	1.1%	0.06 [0.03; 0.08]
Miyazato et al,, 2022 G1	16	457	1.1%	0.04 [0.02; 0.05]
Miyazato et al,, 2022 G2	12	457	1.1%	0.03 [0.01; 0.04]
Nayani et al,, 2023	104	1531	1.1%	0.07 [0.06; 0.08]
Nguyen et al,, 2022	103	605	1.1%	0.17 [0.14; 0.20]
Righi et al,, 2022 G1	0	417	0.5%	0.00 [0.00; 0.00]
Righi et al,, 2022 G2	16	417	1.1%	0.04 [0.02; 0.06]
Silva et al,, 2023	43	1371	1.1%	0.03 [0.02; 0.04]
Vaira et al,, 2022	33	170	1.1%	0.19 [0.13; 0.25]
Wang et al,, 2023	26	1546	1.1%	0.02 [0.01; 0.02]
**Total (95% CI)**		**27697**	**37.4%**	**0.06 [0.04; 0.09]**
Heterogeneity: $Tau^2 = 1.55$; $Chi^2 = 1428.93$, df = 34 (P < 0.0001); $I^2 = 97.6\%$				
**Bias = Moderate**				
Alabsi et al,, 2022 G1	14	100	1.1%	0.14 [0.07; 0.21]
Alabsi et al,, 2022 G2	54	100	1.1%	0.54 [0.44; 0.64]
Aldahleh et al,, 2023	33	261	1.1%	0.13 [0.09; 0.17]
Asakura et al,, 2023 G1	152	4328	1.2%	0.04 [0.03; 0.04]
Asakura et al,, 2023 G2	173	4328	1.2%	0.04 [0.03; 0.05]
Asakura et al,, 2023 G3	80	4328	1.1%	0.02 [0.01; 0.02]
Asakura et al,, 2023 G4	51	4328	1.1%	0.01 [0.01; 0.01]
Bertuccio et al,, 2023	24	323	1.1%	0.07 [0.05; 0.10]
Damanti et al,, 2022	1	176	0.7%	0.01 [0.00; 0.02]
Estrada−Codecido et al,, 2022 G1	4	140	1.0%	0.03 [0.00; 0.06]
Estrada−Codecido et al,, 2022 G2	12	326	1.1%	0.04 [0.02; 0.06]
Fang et al,, 2022	13	843	1.1%	0.02 [0.01; 0.02]
Fernández−de−las−Peñas et al, 2021	38	1142	1.1%	0.03 [0.02; 0.04]
Fernández−de−las−Peñas et al, 2022c G1	6	109	1.0%	0.06 [0.01; 0.10]
Fernández−de−las−Peñas et al, 2022c G2	4	92	1.0%	0.04 [0.00; 0.09]
Ferrucci et al,, 2022 G1	36	76	1.1%	0.47 [0.36; 0.59]
Ferrucci et al,, 2022 G2	7	53	1.1%	0.13 [0.04; 0.22]
Ghanem et al,, 2023	3	37	0.9%	0.08 [0.00; 0.17]
Gherlone et al,, 2021	14	122	1.1%	0.11 [0.06; 0.17]
Hintschich et al,, 2022	97	151	1.1%	0.64 [0.57; 0.72]
Huang et al, 2022 G1	87	1151	1.1%	0.08 [0.06; 0.09]
Huang et al, 2022 G2	35	1188	1.1%	0.03 [0.02; 0.04]
Huang et al, 2022 G3	35	1190	1.1%	0.03 [0.02; 0.04]
Imoto et al,, 2022	54	285	1.1%	0.19 [0.14; 0.23]
Kalak et al,, 2022	10	166	1.1%	0.06 [0.02; 0.10]
Khanafer et al,, 2023	5	178	1.0%	0.03 [0.00; 0.05]
Kaplan et al,, 2021	23	121	1.1%	0.19 [0.12; 0.26]
Kim Y et al,, 2022	3	170	1.0%	0.02 [0.00; 0.04]
Lemhöfer et al,, 2021	16	365	1.1%	0.04 [0.02; 0.06]
Messin et al,, 2021	8	74	1.1%	0.11 [0.04; 0.18]
Olavegogeascoechea et al,, 2022	240	1539	1.2%	0.16 [0.14; 0.17]
Peluso et al,, 2022	12	179	1.1%	0.07 [0.03; 0.10]
Quinones−Moya et al,, 2022	11	64	1.1%	0.17 [0.08; 0.26]
Rass et al,, 2022 G1	110	479	1.1%	0.23 [0.19; 0.27]
Rass et al,, 2022 G2	89	427	1.1%	0.21 [0.17; 0.25]
Sakurada et al,, 2022	19	65	1.1%	0.29 [0.18; 0.40]
Santana et al,, 2021 G1	2	15	0.9%	0.13 [0.00; 0.31]
Santana et al,, 2021 G2	0	15	0.5%	0.00 [0.00; 0.00]
Santana et al,, 2021 G3	4	15	1.0%	0.27 [0.04; 0.49]
Santana et al,, 2021 G4	1	15	0.7%	0.07 [0.00; 0.19]
Shah et al,, 2022	13	300	1.1%	0.04 [0.02; 0.07]
Titze et al,, 2022 G1	22	236	1.1%	0.09 [0.06; 0.13]
Titze et al,, 2022 G2	22	236	1.1%	0.09 [0.06; 0.13]
Vaes et al,, 2021 G1	201	1556	1.2%	0.13 [0.11; 0.15]
Vaes et al,, 2021 G2	190	1005	1.2%	0.19 [0.16; 0.21]
Wong−Chew ta al,, 2022	25	928	1.1%	0.03 [0.02; 0.04]
**Total (95% CI)**		**33325**	**49.0%**	**0.08 [0.06; 0.11]**
Heterogeneity: $Tau^2 = 1.32$; $Chi^2 = 2020.72$, df = 45 (P = 0); $I^2 = 97.8\%$				
**Bias = High**				
AlBahrani et al,, 2023	1	15	0.7%	0.07 [0.00; 0.19]
Bellan et al,, 2022	17	122	1.1%	0.14 [0.08; 0.20]
Biadsee et al,, 2021	25	97	1.1%	0.26 [0.17; 0.34]
Bougea et al,, 2023	25	38	1.1%	0.66 [0.51; 0.81]
Brito−Zerón et al,, 2021	16	132	1.1%	0.12 [0.07; 0.18]
Bussiere et al,, 2022	102	366	1.1%	0.28 [0.23; 0.32]
Howe et al,, 2021	15	63	1.1%	0.24 [0.13; 0.34]
Klein et al,, 2021	7	103	1.1%	0.07 [0.02; 0.12]
Klopfenstein et al,, 2022 G1	2	214	0.9%	0.01 [0.00; 0.02]
Klopfenstein et al,, 2022 G2	62	214	1.1%	0.29 [0.23; 0.35]
Sadat Larijani et al,, 2022 G1	6	82	1.0%	0.07 [0.02; 0.13]
Sadat Larijani et al,, 2022 G2	3	54	1.0%	0.06 [0.00; 0.12]
Van Elst et al,, 2022	133	157	1.1%	0.85 [0.79; 0.90]
**Total (95% CI)**		**1657**	**13.6%**	**0.18 [0.08; 0.34]**
Heterogeneity: $Tau^2 = 2.36$; $Chi^2 = 265.15$, df = 12 (P < 0.0001); $I^2 = 95.5\%$				
**Total (95% CI)**		**62679**	**100.0%**	**0.08 [0.06; 0.10]**
Heterogeneity: $Tau^2 = 1.67$; $Chi^2 = 4228.12$, df = 93 (P = 0); $I^2 = 97.8\%$				
Test for subgroup differences: $Chi^2 = 6.98$, df = 2 (P = 0.0305)				

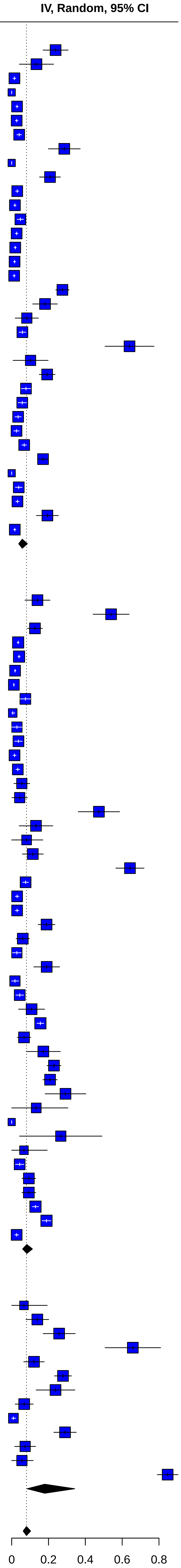

Meta-Analysis of Taste Dysfunction: Forest Plot by Risk of Bias Subgroups

Study or Subgroup	Events	Total	Weight	IV, Random, 95% CI
**Time = 12 to 24 weeks**				
Akova; Gedikli,, 2022	36	151	1.1%	0.24 [0.17; 0.31]
Alabsi et al,, 2022 G1	14	100	1.1%	0.14 [0.07; 0.21]
Alabsi et al,, 2022 G2	54	100	1.1%	0.54 [0.44; 0.64]
AlBahrani et al,, 2023	1	15	0.7%	0.07 [0.00; 0.19]
Aldahleh et al,, 2023	33	261	1.1%	0.13 [0.09; 0.17]
Ali et al,, 2022	7	52	1.1%	0.13 [0.04; 0.23]
AlRadini et al,, 2022	15	1000	1.1%	0.01 [0.01; 0.02]
Arjun et al,, 2022	0	34	0.5%	0.00 [0.00; 0.00]
Asadi−Pooya et al,, 2021 G1	78	2685	1.1%	0.03 [0.02; 0.04]
Asakura et al,, 2023 G1	152	4328	1.2%	0.04 [0.03; 0.04]
Augustin et al,, 2021	39	958	1.1%	0.04 [0.03; 0.05]
Bellan et al,, 2022	17	122	1.1%	0.14 [0.08; 0.20]
Bertuccio et al,, 2023	24	323	1.1%	0.07 [0.05; 0.10]
Biadsee et al,, 2021	25	97	1.1%	0.26 [0.17; 0.34]
Boscolo−Rizzo et al,, 2022b	30	105	1.1%	0.29 [0.20; 0.37]
Boscolo−Rizzo et al,, 2023b	0	294	0.5%	0.00 [0.00; 0.00]
Bougea et al,, 2023	25	38	1.1%	0.66 [0.51; 0.81]
Brito−Zerón et al,, 2021	16	132	1.1%	0.12 [0.07; 0.18]
Bussiere et al,, 2022	102	366	1.1%	0.28 [0.23; 0.32]
Damanti et al,, 2022	1	176	0.7%	0.01 [0.00; 0.02]
Elnadi et al,, 2023	41	197	1.1%	0.21 [0.15; 0.26]
Estrada−Codecido et al,, 2022 G1	4	140	1.0%	0.03 [0.00; 0.06]
Estrada−Codecido et al,, 2022 G2	12	326	1.1%	0.04 [0.02; 0.06]
Fang et al,, 2022	13	843	1.1%	0.02 [0.01; 0.02]
Fernández−de−las−Peñas et al, 2021	38	1142	1.1%	0.03 [0.02; 0.04]
Fernández−de−las−Peñas et al, 2022b	29	614	1.1%	0.05 [0.03; 0.06]
Fernández−de−las−Peñas et al, 2022c G1	6	109	1.0%	0.06 [0.01; 0.10]
Fernández−de−las−Peñas et al, 2022c G2	4	92	1.0%	0.04 [0.00; 0.09]
Fernández−de−las−Peñas et al, 2022d	53	1969	1.1%	0.03 [0.02; 0.03]
Fernández−de−las−Peñas et al, 2023 G1	38	1969	1.1%	0.02 [0.01; 0.03]
Ferrucci et al,, 2022 G1	36	76	1.1%	0.47 [0.36; 0.59]
Förster et al, 2022 G1	162	588	1.1%	0.28 [0.24; 0.31]
Förster et al, 2022 G2	23	127	1.1%	0.18 [0.11; 0.25]
Fornazieri et al,, 2022	6	73	1.0%	0.08 [0.02; 0.15]
Fortunato et al,, 2022	38	653	1.1%	0.06 [0.04; 0.08]
Ghanem et al,, 2023	3	37	0.9%	0.08 [0.00; 0.17]
Gherlone et al,, 2021	14	122	1.1%	0.11 [0.06; 0.17]
Graham et al,, 2021	32	50	1.1%	0.64 [0.51; 0.77]
Gudziol et al,, 2022	4	39	1.0%	0.10 [0.01; 0.20]
Hintschich et al,, 2022	97	151	1.1%	0.64 [0.57; 0.72]
Howe et al,, 2021	15	63	1.1%	0.24 [0.13; 0.34]
Huang et al, 2022 G1	87	1151	1.1%	0.08 [0.06; 0.09]
Imoto et al,, 2022	54	285	1.1%	0.19 [0.14; 0.23]
Jabali et al,, 2022	63	327	1.1%	0.19 [0.15; 0.24]
Kalak et al,, 2022	10	166	1.1%	0.06 [0.02; 0.10]
Khanafer et al,, 2023	5	178	1.0%	0.03 [0.00; 0.05]
Kaplan et al,, 2021	23	121	1.1%	0.19 [0.12; 0.26]
Kim Y et al,, 2022	3	170	1.0%	0.02 [0.00; 0.04]
Klein et al,, 2021	7	103	1.1%	0.07 [0.02; 0.12]
Klopfenstein et al,, 2022 G1	2	214	0.9%	0.01 [0.00; 0.02]
Klopfenstein et al,, 2022 G2	62	214	1.1%	0.29 [0.23; 0.35]
Lapa et al,, 2023 G1	31	400	1.1%	0.08 [0.05; 0.10]
Lapa et al,, 2023 G2	23	400	1.1%	0.06 [0.03; 0.08]
Lemhöfer et al,, 2021	16	365	1.1%	0.04 [0.02; 0.06]
Messin et al,, 2021	8	74	1.1%	0.11 [0.04; 0.18]
Miyazato et al,, 2022 G1	16	457	1.1%	0.04 [0.02; 0.05]
Nayani et al,, 2023	104	1531	1.1%	0.07 [0.06; 0.08]
Nguyen et al,, 2022	103	605	1.1%	0.17 [0.14; 0.20]
Olavegogeascoechea et al,, 2022	240	1539	1.2%	0.16 [0.14; 0.17]
Peluso et al,, 2022	12	179	1.1%	0.07 [0.03; 0.10]
Quinones−Moya et al,, 2022	11	64	1.1%	0.17 [0.08; 0.26]
Rass et al,, 2022 G1	110	479	1.1%	0.23 [0.19; 0.27]
Rass et al,, 2022 G2	89	427	1.1%	0.21 [0.17; 0.25]
Righi et al,, 2022 G1	0	417	0.5%	0.00 [0.00; 0.00]
Righi et al,, 2022 G2	16	417	1.1%	0.04 [0.02; 0.06]
Sadat Larijani et al,, 2022 G1	6	82	1.0%	0.07 [0.02; 0.13]
Sadat Larijani et al,, 2022 G2	3	54	1.0%	0.06 [0.00; 0.12]
Sakurada et al,, 2022	19	65	1.1%	0.29 [0.18; 0.40]
Santana et al,, 2021 G1	2	15	0.9%	0.13 [0.00; 0.31]
Santana et al,, 2021 G3	4	15	1.0%	0.27 [0.04; 0.49]
Shah et al,, 2022	13	300	1.1%	0.04 [0.02; 0.07]
Silva et al,, 2023	43	1371	1.1%	0.03 [0.02; 0.04]
Titze et al,, 2022 G1	22	236	1.1%	0.09 [0.06; 0.13]
Titze et al,, 2022 G2	22	236	1.1%	0.09 [0.06; 0.13]
Vaes et al,, 2021 G1	201	1556	1.2%	0.13 [0.11; 0.15]
Vaes et al,, 2021 G2	190	1005	1.2%	0.19 [0.16; 0.21]
Vaira et al,, 2022	33	170	1.1%	0.19 [0.13; 0.25]
Van Elst et al,, 2022	133	157	1.1%	0.85 [0.79; 0.90]
Wang et al,, 2023	26	1546	1.1%	0.02 [0.01; 0.02]
Wong−Chew ta al,, 2022	25	928	1.1%	0.03 [0.02; 0.04]
**Total (95% CI)**		**38736**	**85.3%**	**0.10 [0.07; 0.12]**
Heterogeneity: $Tau^2 = 1.61$; $Chi^2 = 2954.23$, df = 79 (P = 0); $I^2 = 97.3\%$				
**Time = 24 to 48 weeks**				
Asadi−Pooya et al,, 2021 G2	54	1996	1.1%	0.03 [0.02; 0.03]
Asakura et al,, 2023 G2	173	4328	1.2%	0.04 [0.03; 0.05]
Asakura et al,, 2023 G3	80	4328	1.1%	0.02 [0.01; 0.02]
Fernández−de−las−Peñas et al, 2022a G1	48	1593	1.1%	0.03 [0.02; 0.04]
Fernández−de−las−Peñas et al, 2023 G2	25	1593	1.1%	0.02 [0.01; 0.02]
Ferrucci et al,, 2022 G2	7	53	1.1%	0.13 [0.04; 0.22]
Huang et al, 2022 G2	35	1188	1.1%	0.03 [0.02; 0.04]
Miyazato et al,, 2022 G2	12	457	1.1%	0.03 [0.01; 0.04]
Santana et al,, 2021 G2	0	15	0.5%	0.00 [0.00; 0.00]
Santana et al,, 2021 G4	1	15	0.7%	0.07 [0.00; 0.19]
**Total (95% CI)**		**15566**	**10.2%**	**0.03 [0.02; 0.04]**
Heterogeneity: $Tau^2 = 0.26$; $Chi^2 = 61.69$, df = 9 (P < 0.0001); $I^2 = 85.4\%$				
**Time = more than 48 weeks**				
Asakura et al,, 2023 G4	51	4328	1.1%	0.01 [0.01; 0.01]
Fernández−de−las−Peñas et al, 2022a G2	28	1593	1.1%	0.02 [0.01; 0.02]
Fernández−de−las−Peñas et al, 2023 G3	17	1266	1.1%	0.01 [0.01; 0.02]
Huang et al, 2022 G3	35	1190	1.1%	0.03 [0.02; 0.04]
**Total (95% CI)**		**8377**	**4.5%**	**0.02 [0.01; 0.03]**
Heterogeneity: $Tau^2 = 0.15$; $Chi^2 = 18.59$, df = 3 (P = 0.0003); $I^2 = 83.9\%$				
**Total (95% CI)**		**62679**	**100.0%**	**0.08 [0.06; 0.10]**
Heterogeneity: $Tau^2 = 1.67$; $Chi^2 = 4228.12$, df = 93 (P = 0); $I^2 = 97.8\%$				
Test for subgroup differences: $Chi^2 = 56.93$, df = 2 (P < 0.0001)				

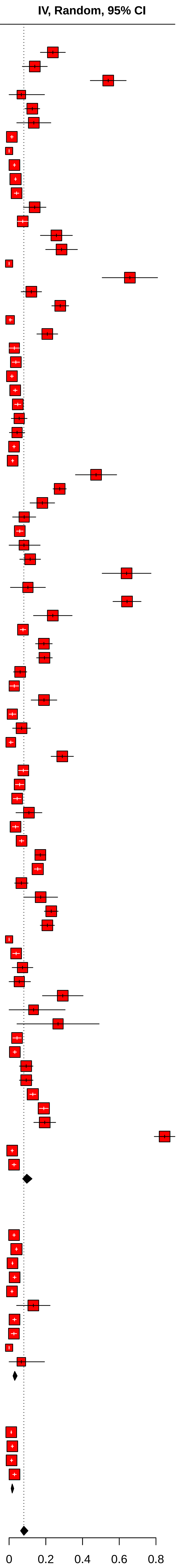

Meta-Analysis of Taste Dysfunction: Forest Plot by Time Subgroups

Study or Subgroup	Events	Total	Weight	IV, Random, 95% CI
**Nsample = Less than 300 patients**				
Akova; Gedikli,, 2022	36	151	1.1%	0.24 [0.17; 0.31]
Alabsi et al,, 2022 G1	14	100	1.1%	0.14 [0.07; 0.21]
Alabsi et al,, 2022 G2	54	100	1.1%	0.54 [0.44; 0.64]
AlBahrani et al,, 2023	1	15	0.7%	0.07 [0.00; 0.19]
Aldahleh et al,, 2023	33	261	1.1%	0.13 [0.09; 0.17]
Ali et al,, 2022	7	52	1.1%	0.13 [0.04; 0.23]
Arjun et al,, 2022	0	34	0.5%	0.00 [0.00; 0.00]
Bellan et al,, 2022	17	122	1.1%	0.14 [0.08; 0.20]
Biadsee et al,, 2021	25	97	1.1%	0.26 [0.17; 0.34]
Boscolo−Rizzo et al,, 2022b	30	105	1.1%	0.29 [0.20; 0.37]
Boscolo−Rizzo et al,, 2023b	0	294	0.5%	0.00 [0.00; 0.00]
Bougea et al,, 2023	25	38	1.1%	0.66 [0.51; 0.81]
Brito−Zerón et al,, 2021	16	132	1.1%	0.12 [0.07; 0.18]
Damanti et al,, 2022	1	176	0.7%	0.01 [0.00; 0.02]
Elnadi et al,, 2023	41	197	1.1%	0.21 [0.15; 0.26]
Estrada−Codecido et al,, 2022 G1	4	140	1.0%	0.03 [0.00; 0.06]
Fernández−de−las−Peñas et al, 2022c G1	6	109	1.0%	0.06 [0.01; 0.10]
Fernández−de−las−Peñas et al, 2022c G2	4	92	1.0%	0.04 [0.00; 0.09]
Ferrucci et al,, 2022 G1	36	76	1.1%	0.47 [0.36; 0.59]
Ferrucci et al,, 2022 G2	7	53	1.1%	0.13 [0.04; 0.22]
Förster et al, 2022 G2	23	127	1.1%	0.18 [0.11; 0.25]
Fornazieri et al,, 2022	6	73	1.0%	0.08 [0.02; 0.15]
Ghanem et al,, 2023	3	37	0.9%	0.08 [0.00; 0.17]
Gherlone et al,, 2021	14	122	1.1%	0.11 [0.06; 0.17]
Graham et al,, 2021	32	50	1.1%	0.64 [0.51; 0.77]
Gudziol et al,, 2022	4	39	1.0%	0.10 [0.01; 0.20]
Hintschich et al,, 2022	97	151	1.1%	0.64 [0.57; 0.72]
Howe et al,, 2021	15	63	1.1%	0.24 [0.13; 0.34]
Imoto et al,, 2022	54	285	1.1%	0.19 [0.14; 0.23]
Kalak et al,, 2022	10	166	1.1%	0.06 [0.02; 0.10]
Khanafer et al,, 2023	5	178	1.0%	0.03 [0.00; 0.05]
Kaplan et al,, 2021	23	121	1.1%	0.19 [0.12; 0.26]
Kim Y et al,, 2022	3	170	1.0%	0.02 [0.00; 0.04]
Klein et al,, 2021	7	103	1.1%	0.07 [0.02; 0.12]
Klopfenstein et al,, 2022 G1	2	214	0.9%	0.01 [0.00; 0.02]
Klopfenstein et al,, 2022 G2	62	214	1.1%	0.29 [0.23; 0.35]
Messin et al,, 2021	8	74	1.1%	0.11 [0.04; 0.18]
Peluso et al,, 2022	12	179	1.1%	0.07 [0.03; 0.10]
Quinones−Moya et al,, 2022	11	64	1.1%	0.17 [0.08; 0.26]
Sadat Larijani et al,, 2022 G1	6	82	1.0%	0.07 [0.02; 0.13]
Sadat Larijani et al,, 2022 G2	3	54	1.0%	0.06 [0.00; 0.12]
Sakurada et al,, 2022	19	65	1.1%	0.29 [0.18; 0.40]
Santana et al,, 2021 G1	2	15	0.9%	0.13 [0.00; 0.31]
Santana et al,, 2021 G2	0	15	0.5%	0.00 [0.00; 0.00]
Santana et al,, 2021 G3	4	15	1.0%	0.27 [0.04; 0.49]
Santana et al,, 2021 G4	1	15	0.7%	0.07 [0.00; 0.19]
Titze et al,, 2022 G1	22	236	1.1%	0.09 [0.06; 0.13]
Titze et al,, 2022 G2	22	236	1.1%	0.09 [0.06; 0.13]
Vaira et al,, 2022	33	170	1.1%	0.19 [0.13; 0.25]
Van Elst et al,, 2022	133	157	1.1%	0.85 [0.79; 0.90]
**Total (95% CI)**		**5834**	**50.7%**	**0.14 [0.10; 0.19]**
Heterogeneity: Tau^2 = 1.60; Chi^2 = 788.06, df = 49 (P < 0.0001); I^2 = 93.8%				
**Nsample = More than 300 patients**				
AlRadini et al,, 2022	15	1000	1.1%	0.01 [0.01; 0.02]
Asadi−Pooya et al,, 2021 G1	78	2685	1.1%	0.03 [0.02; 0.04]
Asadi−Pooya et al,, 2021 G2	54	1996	1.1%	0.03 [0.02; 0.03]
Asakura et al,, 2023 G1	152	4328	1.2%	0.04 [0.03; 0.04]
Asakura et al,, 2023 G2	173	4328	1.2%	0.04 [0.03; 0.05]
Asakura et al,, 2023 G3	80	4328	1.1%	0.02 [0.01; 0.02]
Asakura et al,, 2023 G4	51	4328	1.1%	0.01 [0.01; 0.01]
Augustin et al,, 2021	39	958	1.1%	0.04 [0.03; 0.05]
Bertuccio et al,, 2023	24	323	1.1%	0.07 [0.05; 0.10]
Bussiere et al,, 2022	102	366	1.1%	0.28 [0.23; 0.32]
Estrada−Codecido et al,, 2022 G2	12	326	1.1%	0.04 [0.02; 0.06]
Fang et al,, 2022	13	843	1.1%	0.02 [0.01; 0.02]
Fernández−de−las−Peñas et al, 2021	38	1142	1.1%	0.03 [0.02; 0.04]
Fernández−de−las−Peñas et al, 2022a G1	48	1593	1.1%	0.03 [0.02; 0.04]
Fernández−de−las−Peñas et al, 2022a G2	28	1593	1.1%	0.02 [0.01; 0.02]
Fernández−de−las−Peñas et al, 2022b	29	614	1.1%	0.05 [0.03; 0.06]
Fernández−de−las−Peñas et al, 2022d	53	1969	1.1%	0.03 [0.02; 0.03]
Fernández−de−las−Peñas et al, 2023 G1	38	1969	1.1%	0.02 [0.01; 0.03]
Fernández−de−las−Peñas et al, 2023 G2	25	1593	1.1%	0.02 [0.01; 0.02]
Fernández−de−las−Peñas et al, 2023 G3	17	1266	1.1%	0.01 [0.01; 0.02]
Förster et al, 2022 G1	162	588	1.1%	0.28 [0.24; 0.31]
Fortunato et al,, 2022	38	653	1.1%	0.06 [0.04; 0.08]
Huang et al, 2022 G1	87	1151	1.1%	0.08 [0.06; 0.09]
Huang et al, 2022 G2	35	1188	1.1%	0.03 [0.02; 0.04]
Huang et al, 2022 G3	35	1190	1.1%	0.03 [0.02; 0.04]
Jabali et al,, 2022	63	327	1.1%	0.19 [0.15; 0.24]
Lapa et al,, 2023 G1	31	400	1.1%	0.08 [0.05; 0.10]
Lapa et al,, 2023 G2	23	400	1.1%	0.06 [0.03; 0.08]
Lemhöfer et al,, 2021	16	365	1.1%	0.04 [0.02; 0.06]
Miyazato et al,, 2022 G1	16	457	1.1%	0.04 [0.02; 0.05]
Miyazato et al,, 2022 G2	12	457	1.1%	0.03 [0.01; 0.04]
Nayani et al,, 2023	104	1531	1.1%	0.07 [0.06; 0.08]
Nguyen et al,, 2022	103	605	1.1%	0.17 [0.14; 0.20]
Olavegogeascoechea et al,, 2022	240	1539	1.2%	0.16 [0.14; 0.17]
Rass et al,, 2022 G1	110	479	1.1%	0.23 [0.19; 0.27]
Rass et al,, 2022 G2	89	427	1.1%	0.21 [0.17; 0.25]
Righi et al,, 2022 G1	0	417	0.5%	0.00 [0.00; 0.00]
Righi et al,, 2022 G2	16	417	1.1%	0.04 [0.02; 0.06]
Shah et al,, 2022	13	300	1.1%	0.04 [0.02; 0.07]
Silva et al,, 2023	43	1371	1.1%	0.03 [0.02; 0.04]
Vaes et al,, 2021 G1	201	1556	1.2%	0.13 [0.11; 0.15]
Vaes et al,, 2021 G2	190	1005	1.2%	0.19 [0.16; 0.21]
Wang et al,, 2023	26	1546	1.1%	0.02 [0.01; 0.02]
Wong−Chew ta al,, 2022	25	928	1.1%	0.03 [0.02; 0.04]
**Total (95% CI)**		**56845**	**49.3%**	**0.05 [0.03; 0.06]**
Heterogeneity: Tau^2 = 0.97; Chi^2 = 2590.33, df = 43 (P = 0); I^2 = 98.3%				
**Total (95% CI)**		**62679**	**100.0%**	**0.08 [0.06; 0.10]**
Heterogeneity: Tau^2 = 1.67; Chi^2 = 4228.12, df = 93 (P = 0); I^2 = 97.8%				
Test for subgroup differences: Chi^2 = 23.08, df = 1 (P < 0.0001)				

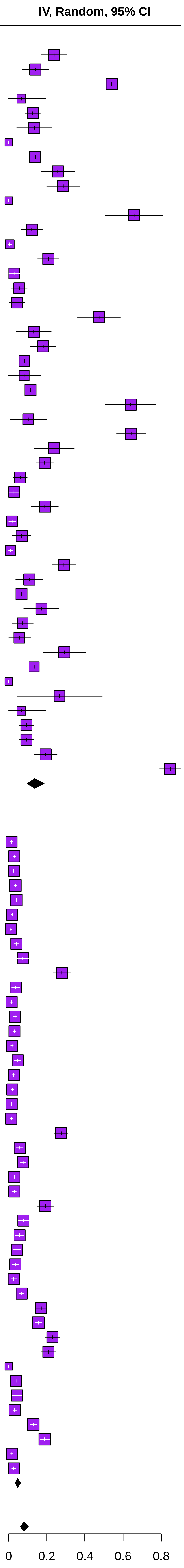

Meta-Analysis of Taste Dysfunction: Forest Plot by Sample Size Subgroups

Study or Subgroup	Events	Total	Weight	IV, Random, 95% CI
**DisfunctionType = Ageusia**				
Akova; Gedikli,, 2022	36	151	1.1%	0.24 [0.17; 0.31]
Alabsi et al,, 2022 G1	14	100	1.1%	0.14 [0.07; 0.21]
Ali et al,, 2022	7	52	1.1%	0.13 [0.04; 0.23]
AlRadini et al,, 2022	15	1000	1.1%	0.01 [0.01; 0.02]
Asadi−Pooya et al,, 2021 G1	78	2685	1.1%	0.03 [0.02; 0.04]
Asadi−Pooya et al,, 2021 G2	54	1996	1.1%	0.03 [0.02; 0.03]
Asakura et al,, 2023 G1	152	4328	1.2%	0.04 [0.03; 0.04]
Asakura et al,, 2023 G2	173	4328	1.2%	0.04 [0.03; 0.05]
Asakura et al,, 2023 G3	80	4328	1.1%	0.02 [0.01; 0.02]
Asakura et al,, 2023 G4	51	4328	1.1%	0.01 [0.01; 0.01]
Augustin et al,, 2021	39	958	1.1%	0.04 [0.03; 0.05]
Elnadi et al,, 2023	41	197	1.1%	0.21 [0.15; 0.26]
Fernández−de−las−Peñas et al, 2021	38	1142	1.1%	0.03 [0.02; 0.04]
Fernández−de−las−Peñas et al, 2022a G1	48	1593	1.1%	0.03 [0.02; 0.04]
Fernández−de−las−Peñas et al, 2022a G2	28	1593	1.1%	0.02 [0.01; 0.02]
Fernández−de−las−Peñas et al, 2022b	29	614	1.1%	0.05 [0.03; 0.06]
Fernández−de−las−Peñas et al, 2022c G1	6	109	1.0%	0.06 [0.01; 0.10]
Fernández−de−las−Peñas et al, 2022c G2	4	92	1.0%	0.04 [0.00; 0.09]
Fernández−de−las−Peñas et al, 2022d	53	1969	1.1%	0.03 [0.02; 0.03]
Fernández−de−las−Peñas et al, 2023 G1	38	1969	1.1%	0.02 [0.01; 0.03]
Fernández−de−las−Peñas et al, 2023 G2	25	1593	1.1%	0.02 [0.01; 0.02]
Fernández−de−las−Peñas et al, 2023 G3	17	1266	1.1%	0.01 [0.01; 0.02]
Förster et al, 2022 G1	162	588	1.1%	0.28 [0.24; 0.31]
Förster et al, 2022 G2	23	127	1.1%	0.18 [0.11; 0.25]
Fortunato et al,, 2022	38	653	1.1%	0.06 [0.04; 0.08]
Ghanem et al,, 2023	3	37	0.9%	0.08 [0.00; 0.17]
Gudziol et al,, 2022	4	39	1.0%	0.10 [0.01; 0.20]
Howe et al,, 2021	15	63	1.1%	0.24 [0.13; 0.34]
Huang et al, 2022 G1	87	1151	1.1%	0.08 [0.06; 0.09]
Huang et al, 2022 G2	35	1188	1.1%	0.03 [0.02; 0.04]
Huang et al, 2022 G3	35	1190	1.1%	0.03 [0.02; 0.04]
Jabali et al,, 2022	63	327	1.1%	0.19 [0.15; 0.24]
Kalak et al,, 2022	10	166	1.1%	0.06 [0.02; 0.10]
Khanafer et al,, 2023	5	178	1.0%	0.03 [0.00; 0.05]
Kaplan et al,, 2021	23	121	1.1%	0.19 [0.12; 0.26]
Kim Y et al,, 2022	3	170	1.0%	0.02 [0.00; 0.04]
Klopfenstein et al,, 2022 G1	2	214	0.9%	0.01 [0.00; 0.02]
Lapa et al,, 2023 G1	31	400	1.1%	0.08 [0.05; 0.10]
Lapa et al,, 2023 G2	23	400	1.1%	0.06 [0.03; 0.08]
Messin et al,, 2021	8	74	1.1%	0.11 [0.04; 0.18]
Nayani et al,, 2023	104	1531	1.1%	0.07 [0.06; 0.08]
Righi et al,, 2022 G1	0	417	0.5%	0.00 [0.00; 0.00]
Sadat Larijani et al,, 2022 G1	6	82	1.0%	0.07 [0.02; 0.13]
Sadat Larijani et al,, 2022 G2	3	54	1.0%	0.06 [0.00; 0.12]
Santana et al,, 2021 G1	2	15	0.9%	0.13 [0.00; 0.31]
Santana et al,, 2021 G2	0	15	0.5%	0.00 [0.00; 0.00]
Shah et al,, 2022	13	300	1.1%	0.04 [0.02; 0.07]
Silva et al,, 2023	43	1371	1.1%	0.03 [0.02; 0.04]
Titze et al,, 2022 G1	22	236	1.1%	0.09 [0.06; 0.13]
Vaes et al,, 2021 G1	201	1556	1.2%	0.13 [0.11; 0.15]
Vaes et al,, 2021 G2	190	1005	1.2%	0.19 [0.16; 0.21]
**Total (95% CI)**		**50059**	**54.8%**	**0.05 [0.04; 0.07]**
Heterogeneity: $Tau^2 = 0.91$; $Chi^2 = 1896.35$, df = 50 (P = 0); $I^2 = 97.4\%$				
**DisfunctionType = Dysgeusia**				
Alabsi et al,, 2022 G2	54	100	1.1%	0.54 [0.44; 0.64]
AlBahrani et al,, 2023	1	15	0.7%	0.07 [0.00; 0.19]
Aldahleh et al,, 2023	33	261	1.1%	0.13 [0.09; 0.17]
Arjun et al,, 2022	0	34	0.5%	0.00 [0.00; 0.00]
Bellan et al,, 2022	17	122	1.1%	0.14 [0.08; 0.20]
Bertuccio et al,, 2023	24	323	1.1%	0.07 [0.05; 0.10]
Biadsee et al,, 2021	25	97	1.1%	0.26 [0.17; 0.34]
Boscolo−Rizzo et al,, 2023b	0	294	0.5%	0.00 [0.00; 0.00]
Bougea et al,, 2023	25	38	1.1%	0.66 [0.51; 0.81]
Brito−Zerón et al,, 2021	16	132	1.1%	0.12 [0.07; 0.18]
Bussiere et al,, 2022	102	366	1.1%	0.28 [0.23; 0.32]
Damanti et al,, 2022	1	176	0.7%	0.01 [0.00; 0.02]
Estrada−Codecido et al,, 2022 G1	4	140	1.0%	0.03 [0.00; 0.06]
Estrada−Codecido et al,, 2022 G2	12	326	1.1%	0.04 [0.02; 0.06]
Fang et al,, 2022	13	843	1.1%	0.02 [0.01; 0.02]
Ferrucci et al,, 2022 G1	36	76	1.1%	0.47 [0.36; 0.59]
Ferrucci et al,, 2022 G2	7	53	1.1%	0.13 [0.04; 0.22]
Fornazieri et al,, 2022	6	73	1.0%	0.08 [0.02; 0.15]
Gherlone et al,, 2021	14	122	1.1%	0.11 [0.06; 0.17]
Graham et al,, 2021	32	50	1.1%	0.64 [0.51; 0.77]
Imoto et al,, 2022	54	285	1.1%	0.19 [0.14; 0.23]
Klein et al,, 2021	7	103	1.1%	0.07 [0.02; 0.12]
Klopfenstein et al,, 2022 G2	62	214	1.1%	0.29 [0.23; 0.35]
Lemhöfer et al,, 2021	16	365	1.1%	0.04 [0.02; 0.06]
Miyazato et al,, 2022 G1	16	457	1.1%	0.04 [0.02; 0.05]
Miyazato et al,, 2022 G2	12	457	1.1%	0.03 [0.01; 0.04]
Nguyen et al,, 2022	103	605	1.1%	0.17 [0.14; 0.20]
Olavegogeascoechea et al,, 2022	240	1539	1.2%	0.16 [0.14; 0.17]
Peluso et al,, 2022	12	179	1.1%	0.07 [0.03; 0.10]
Quinones−Moya et al,, 2022	11	64	1.1%	0.17 [0.08; 0.26]
Rass et al,, 2022 G1	110	479	1.1%	0.23 [0.19; 0.27]
Rass et al,, 2022 G2	89	427	1.1%	0.21 [0.17; 0.25]
Righi et al,, 2022 G2	16	417	1.1%	0.04 [0.02; 0.06]
Sakurada et al,, 2022	19	65	1.1%	0.29 [0.18; 0.40]
Titze et al,, 2022 G2	22	236	1.1%	0.09 [0.06; 0.13]
Vaira et al,, 2022	33	170	1.1%	0.19 [0.13; 0.25]
Van Elst et al,, 2022	133	157	1.1%	0.85 [0.79; 0.90]
Wang et al,, 2023	26	1546	1.1%	0.02 [0.01; 0.02]
Wong−Chew ta al,, 2022	25	928	1.1%	0.03 [0.02; 0.04]
**Total (95% CI)**		**12334**	**41.3%**	**0.12 [0.08; 0.17]**
Heterogeneity: $Tau^2 = 2.06$; $Chi^2 = 1162.26$, df = 38 (P < 0.0001); $I^2 = 96.7\%$				
**DisfunctionType = Hypogeusia**				
Boscolo−Rizzo et al,, 2022b	30	105	1.1%	0.29 [0.20; 0.37]
Hintschich et al,, 2022	97	151	1.1%	0.64 [0.57; 0.72]
Santana et al,, 2021 G3	4	15	1.0%	0.27 [0.04; 0.49]
Santana et al,, 2021 G4	1	15	0.7%	0.07 [0.00; 0.19]
**Total (95% CI)**		**286**	**3.9%**	**0.32 [0.13; 0.60]**
Heterogeneity: $Tau^2 = 1.15$; $Chi^2 = 39.02$, df = 3 (P < 0.0001); $I^2 = 92.3\%$				
**Total (95% CI)**		**62679**	**100.0%**	**0.08 [0.06; 0.10]**
Heterogeneity: $Tau^2 = 1.67$; $Chi^2 = 4228.12$, df = 93 (P = 0); $I^2 = 97.8\%$				
Test for subgroup differences: $Chi^2 = 18.80$, df = 2 (P < 0.0001)				

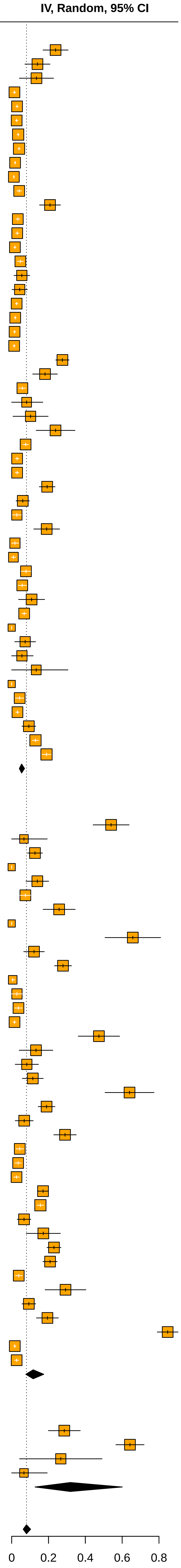

Meta-Analysis of Taste Dysfunction: Forest Plot by Type of Taste Dysfunction Subgroups

Study or Subgroup	Events	Total	Weight	IV, Random, 95% CI
**Bias = Moderate**				
Akinci., 2022	14	85	2.4%	0.16 [0.09; 0.24]
Boscolo−Rizzo et al., 2022c G1	53	174	2.5%	0.30 [0.24; 0.37]
Boscolo−Rizzo et al., 2022c G2	36	174	2.5%	0.21 [0.15; 0.27]
Boscolo−Rizzo et al., 2023b	3	294	1.9%	0.01 [0.00; 0.02]
Brunvoll et al., 2023 G1	62	360	2.5%	0.17 [0.13; 0.21]
Brunvoll et al., 2023 G2	151	1060	2.6%	0.14 [0.12; 0.16]
Caspersen et al., 2021 G1	28	170	2.5%	0.16 [0.11; 0.22]
Caspersen et al., 2021 G2	128	583	2.6%	0.22 [0.19; 0.25]
Chudzik et al., 2022	98	2218	2.6%	0.04 [0.04; 0.05]
Desgranges et al., 2022 G1	64	190	2.5%	0.34 [0.27; 0.40]
Desgranges et al., 2022 G2	22	102	2.4%	0.22 [0.14; 0.30]
Desgranges et al., 2022 G3	21	126	2.4%	0.17 [0.10; 0.23]
Feter et al., 2023	66	237	2.5%	0.28 [0.22; 0.34]
Gottlieb et al., 2023	95	4152	2.6%	0.02 [0.02; 0.03]
Grisanti et al., 2022	86	508	2.5%	0.17 [0.14; 0.20]
Heeney et al., 2023	27	62	2.4%	0.44 [0.31; 0.56]
Kalak et al., 2022	65	156	2.5%	0.42 [0.34; 0.49]
Larsson et al., 2022	551	1584	2.6%	0.35 [0.32; 0.37]
Lindahl et al., 2022	31	101	2.5%	0.31 [0.22; 0.40]
Messin et al., 2021	21	74	2.4%	0.28 [0.18; 0.39]
Nordvig et al., 2023	40	530	2.5%	0.08 [0.05; 0.10]
Pegehin et al., 2021	62	596	2.5%	0.10 [0.08; 0.13]
Peluso et al., 2022 G1	5	143	2.1%	0.03 [0.00; 0.07]
Peluso et al., 2022 G2	12	93	2.4%	0.13 [0.06; 0.20]
Peters et al., 2022	535	1406	2.6%	0.38 [0.36; 0.41]
Schambeck et al., 2021	14	44	2.3%	0.32 [0.18; 0.46]
Schambeck et al., 2022	12	44	2.3%	0.27 [0.14; 0.40]
Scherlinger et al., 2021	3	30	1.9%	0.10 [0.00; 0.21]
Seller et al., 2023	14	110	2.4%	0.13 [0.06; 0.19]
Shafiee et al., 2022	1	3	1.0%	0.33 [0.00; 0.87]
**Total (95% CI)**		**15409**	**71.6%**	**0.17 [0.13; 0.23]**
Heterogeneity: $Tau^2 = 0.90$; $Chi^2 = 1531.63$, df = 29 ($P < 0.0001$); $I^2 = 98.1\%$				
**Bias = Low**				
Boscolo−Rizzo et al., 2023a G1	62	93	2.5%	0.67 [0.57; 0.76]
Boscolo−Rizzo et al., 2023a G2	18	93	2.4%	0.19 [0.11; 0.27]
Duwel et al., 2023 G1	1	13	1.2%	0.08 [0.00; 0.22]
Duwel et al., 2023 G2	0	30	0.9%	0.00 [0.00; 0.00]
Duwel et al., 2023 G3	3	100	1.9%	0.03 [0.00; 0.06]
Duwel et al., 2023 G4	7	79	2.2%	0.09 [0.03; 0.15]
Fortunato et al., 2022	31	653	2.5%	0.05 [0.03; 0.06]
Lombardo et al. 2021	84	303	2.5%	0.28 [0.23; 0.33]
Romero−Duarte et al., 2021	57	797	2.5%	0.07 [0.05; 0.09]
Thi Khanh et al., 2023	300	3708	2.6%	0.08 [0.07; 0.09]
van Zon et al., 2023	190	624	2.6%	0.30 [0.27; 0.34]
**Total (95% CI)**		**6493**	**23.9%**	**0.13 [0.06; 0.24]**
Heterogeneity: $Tau^2 = 1.52$; $Chi^2 = 493.79$, df = 10 ($P < 0.0001$); $I^2 = 98\%$				
**Bias = High**				
Chaumont et al., 2022	23	60	2.4%	0.38 [0.26; 0.51]
Seang et al., 2022	6	39	2.2%	0.15 [0.04; 0.27]
**Total (95% CI)**		**99**	**4.6%**	**0.26 [0.10; 0.54]**
Heterogeneity: $Tau^2 = 0.62$; $Chi^2 = 5.65$, df = 1 ($P = 0.0175$); $I^2 = 82.3\%$				
**Total (95% CI)**		**22001**	**100.0%**	**0.17 [0.13; 0.21]**
Heterogeneity: $Tau^2 = 1.01$; $Chi^2 = 2170.12$, df = 42 ($P = 0$); $I^2 = 98.1\%$				
Test for subgroup differences: $Chi^2 = 1.54$, df = 2 ($P = 0.4621$)				

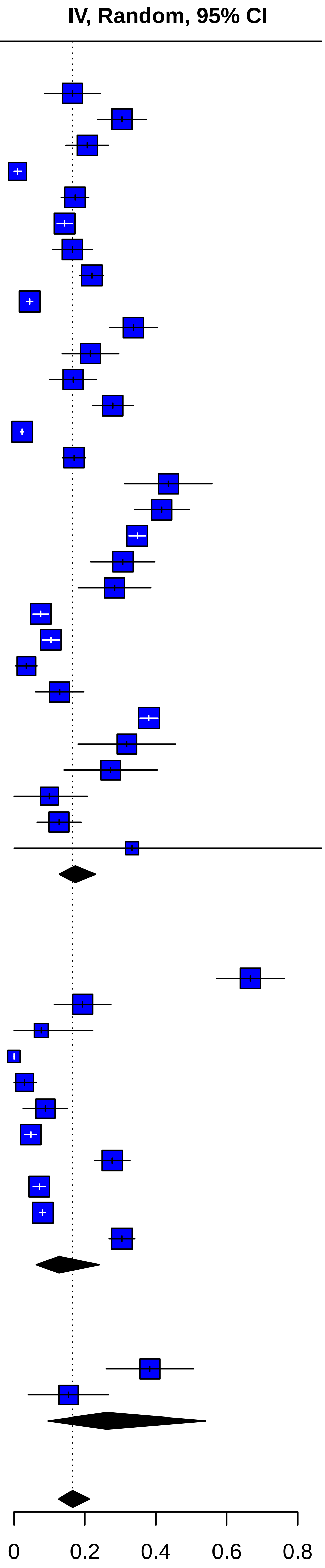

Meta-Analysis of Taste and Smell Dysfunction: Forest Plot by Risk of Bias Subgroups

Study or Subgroup	Events	Total	Weight	IV, Random, 95% CI
**Time = 12 to 24 weeks**				
Akinci., 2022	14	85	2.4%	0.16 [0.09; 0.24]
Boscolo−Rizzo et al., 2023b	3	294	1.9%	0.01 [0.00; 0.02]
Brunvoll et al., 2023 G1	62	360	2.5%	0.17 [0.13; 0.21]
Brunvoll et al., 2023 G2	151	1060	2.6%	0.14 [0.12; 0.16]
Caspersen et al., 2021 G1	28	170	2.5%	0.16 [0.11; 0.22]
Caspersen et al., 2021 G2	128	583	2.6%	0.22 [0.19; 0.25]
Chaumont et al., 2022	23	60	2.4%	0.38 [0.26; 0.51]
Chudzik et al., 2022	98	2218	2.6%	0.04 [0.04; 0.05]
Desgranges et al., 2022 G1	64	190	2.5%	0.34 [0.27; 0.40]
Desgranges et al., 2022 G2	22	102	2.4%	0.22 [0.14; 0.30]
Duwel et al., 2023 G1	1	13	1.2%	0.08 [0.00; 0.22]
Duwel et al., 2023 G2	0	30	0.9%	0.00 [0.00; 0.00]
Fortunato et al., 2022	31	653	2.5%	0.05 [0.03; 0.06]
Feter et al., 2023	66	237	2.5%	0.28 [0.22; 0.34]
Gottlieb et al., 2023	95	4152	2.6%	0.02 [0.02; 0.03]
Grisanti et al., 2022	86	508	2.5%	0.17 [0.14; 0.20]
Heeney et al., 2023	27	62	2.4%	0.44 [0.31; 0.56]
Kalak et al., 2022	65	156	2.5%	0.42 [0.34; 0.49]
Larsson et al., 2022	551	1584	2.6%	0.35 [0.32; 0.37]
Lindahl et al., 2022	31	101	2.5%	0.31 [0.22; 0.40]
Lombardo et al. 2021	84	303	2.5%	0.28 [0.23; 0.33]
Messin et al., 2021	21	74	2.4%	0.28 [0.18; 0.39]
Nordvig et al., 2023	40	530	2.5%	0.08 [0.05; 0.10]
Pegehin et al., 2021	62	596	2.5%	0.10 [0.08; 0.13]
Peluso et al., 2022 G1	5	143	2.1%	0.03 [0.00; 0.07]
Peters et al., 2022	535	1406	2.6%	0.38 [0.36; 0.41]
Romero−Duarte et al., 2021	57	797	2.5%	0.07 [0.05; 0.09]
Schambeck et al., 2021	14	44	2.3%	0.32 [0.18; 0.46]
Scherlinger et al., 2021	3	30	1.9%	0.10 [0.00; 0.21]
Seang et al., 2022	6	39	2.2%	0.15 [0.04; 0.27]
Seller et al., 2023	14	110	2.4%	0.13 [0.06; 0.19]
Shafiee et al., 2022	1	3	1.0%	0.33 [0.00; 0.87]
Thi Khanh et al., 2023	300	3708	2.6%	0.08 [0.07; 0.09]
van Zon et al., 2023	190	624	2.6%	0.30 [0.27; 0.34]
**Total (95% CI)**		**21025**	**78.8%**	**0.16 [0.12; 0.21]**
Heterogeneity: $Tau^2 = 1.01$; $Chi^2 = 2039.93$, df = 33 (P = 0); $I^2 = 98.4\%$				
**Time = 24 to 48 weeks**				
Boscolo−Rizzo et al., 2022c G1	53	174	2.5%	0.30 [0.24; 0.37]
Boscolo−Rizzo et al., 2023a G1	62	93	2.5%	0.67 [0.57; 0.76]
Desgranges et al., 2022 G3	21	126	2.4%	0.17 [0.10; 0.23]
Duwel et al., 2023 G3	3	100	1.9%	0.03 [0.00; 0.06]
Peluso et al., 2022 G2	12	93	2.4%	0.13 [0.06; 0.20]
**Total (95% CI)**		**586**	**11.7%**	**0.20 [0.06; 0.48]**
Heterogeneity: $Tau^2 = 2.09$; $Chi^2 = 91.12$, df = 4 (P < 0.0001); $I^2 = 95.6\%$				
**Time = More than 48 weeks**				
Boscolo−Rizzo et al., 2022c G2	36	174	2.5%	0.21 [0.15; 0.27]
Boscolo−Rizzo et al., 2023a G2	18	93	2.4%	0.19 [0.11; 0.27]
Duwel et al., 2023 G4	7	79	2.2%	0.09 [0.03; 0.15]
Schambeck et al., 2022	12	44	2.3%	0.27 [0.14; 0.40]
**Total (95% CI)**		**390**	**9.5%**	**0.19 [0.13; 0.26]**
Heterogeneity: $Tau^2 = 0.13$; $Chi^2 = 7.14$, df = 3 (P = 0.0675); $I^2 = 58\%$				
**Total (95% CI)**		**22001**	**100.0%**	**0.17 [0.13; 0.21]**
Heterogeneity: $Tau^2 = 1.01$; $Chi^2 = 2170.12$, df = 42 (P = 0); $I^2 = 98.1\%$				
Test for subgroup differences: $Chi^2 = 0.62$, df = 2 (P = 0.7340)				

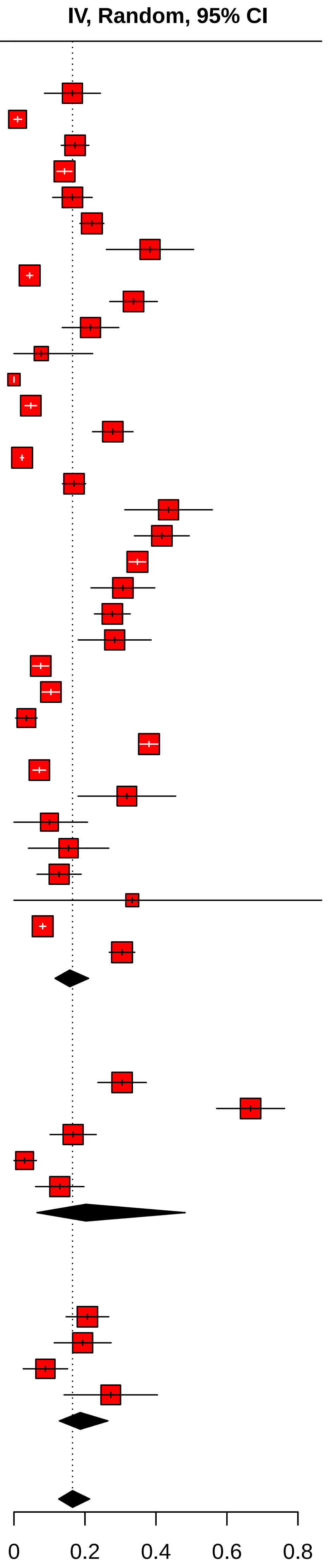

Meta-Analysis of Taste and Smell Dysfunction: Forest Plot by Time Subgroups

Study or Subgroup	Events	Total	Weight	IV, Random, 95% CI
**Nsample = Less than 500 patientes**				
Akinci., 2022	14	85	2.4%	0.16 [0.09; 0.24]
Boscolo−Rizzo et al., 2022c G1	53	174	2.5%	0.30 [0.24; 0.37]
Boscolo−Rizzo et al., 2022c G2	36	174	2.5%	0.21 [0.15; 0.27]
Boscolo−Rizzo et al., 2023a G1	62	93	2.5%	0.67 [0.57; 0.76]
Boscolo−Rizzo et al., 2023a G2	18	93	2.4%	0.19 [0.11; 0.27]
Boscolo−Rizzo et al., 2023b	3	294	1.9%	0.01 [0.00; 0.02]
Brunvoll et al., 2023 G1	62	360	2.5%	0.17 [0.13; 0.21]
Caspersen et al., 2021 G1	28	170	2.5%	0.16 [0.11; 0.22]
Chaumont et al., 2022	23	60	2.4%	0.38 [0.26; 0.51]
Desgranges et al., 2022 G1	64	190	2.5%	0.34 [0.27; 0.40]
Desgranges et al., 2022 G2	22	102	2.4%	0.22 [0.14; 0.30]
Desgranges et al., 2022 G3	21	126	2.4%	0.17 [0.10; 0.23]
Duwel et al., 2023 G1	1	13	1.2%	0.08 [0.00; 0.22]
Duwel et al., 2023 G2	0	30	0.9%	0.00 [0.00; 0.00]
Duwel et al., 2023 G3	3	100	1.9%	0.03 [0.00; 0.06]
Duwel et al., 2023 G4	7	79	2.2%	0.09 [0.03; 0.15]
Feter et al., 2023	66	237	2.5%	0.28 [0.22; 0.34]
Heeney et al., 2023	27	62	2.4%	0.44 [0.31; 0.56]
Kalak et al., 2022	65	156	2.5%	0.42 [0.34; 0.49]
Lindahl et al., 2022	31	101	2.5%	0.31 [0.22; 0.40]
Lombardo et al. 2021	84	303	2.5%	0.28 [0.23; 0.33]
Messin et al., 2021	21	74	2.4%	0.28 [0.18; 0.39]
Peluso et al., 2022 G1	5	143	2.1%	0.03 [0.00; 0.07]
Peluso et al., 2022 G2	12	93	2.4%	0.13 [0.06; 0.20]
Schambeck et al., 2021	14	44	2.3%	0.32 [0.18; 0.46]
Schambeck et al., 2022	12	44	2.3%	0.27 [0.14; 0.40]
Scherlinger et al., 2021	3	30	1.9%	0.10 [0.00; 0.21]
Seang et al., 2022	6	39	2.2%	0.15 [0.04; 0.27]
Seller et al., 2023	14	110	2.4%	0.13 [0.06; 0.19]
Shafiee et al., 2022	1	3	1.0%	0.33 [0.00; 0.87]
**Total (95% CI)**		**3582**	**66.8%**	**0.19 [0.14; 0.26]**
Heterogeneity: $Tau^2 = 0.88$; $Chi^2 = 262.15$, df = 29 (P < 0.0001); $I^2 = 88.9\%$				
**Nsample = More than 500 patients**				
Brunvoll et al., 2023 G2	151	1060	2.6%	0.14 [0.12; 0.16]
Caspersen et al., 2021 G2	128	583	2.6%	0.22 [0.19; 0.25]
Chudzik et al., 2022	98	2218	2.6%	0.04 [0.04; 0.05]
Fortunato et al., 2022	31	653	2.5%	0.05 [0.03; 0.06]
Gottlieb et al., 2023	95	4152	2.6%	0.02 [0.02; 0.03]
Grisanti et al., 2022	86	508	2.5%	0.17 [0.14; 0.20]
Larsson et al., 2022	551	1584	2.6%	0.35 [0.32; 0.37]
Nordvig et al., 2023	40	530	2.5%	0.08 [0.05; 0.10]
Pegehin et al., 2021	62	596	2.5%	0.10 [0.08; 0.13]
Peters et al., 2022	535	1406	2.6%	0.38 [0.36; 0.41]
Romero−Duarte et al., 2021	57	797	2.5%	0.07 [0.05; 0.09]
Thi Khanh et al., 2023	300	3708	2.6%	0.08 [0.07; 0.09]
van Zon et al., 2023	190	624	2.6%	0.30 [0.27; 0.34]
**Total (95% CI)**		**18419**	**33.2%**	**0.12 [0.07; 0.19]**
Heterogeneity: $Tau^2 = 1.02$; $Chi^2 = 1816.88$, df = 12 (P = 0); $I^2 = 99.3\%$				
**Total (95% CI)**		**22001**	**100.0%**	**0.17 [0.13; 0.21]**
Heterogeneity: $Tau^2 = 1.01$; $Chi^2 = 2170.12$, df = 42 (P = 0); $I^2 = 98.1\%$				
Test for subgroup differences: $Chi^2 = 2.95$, df = 1 (P = 0.0857)				

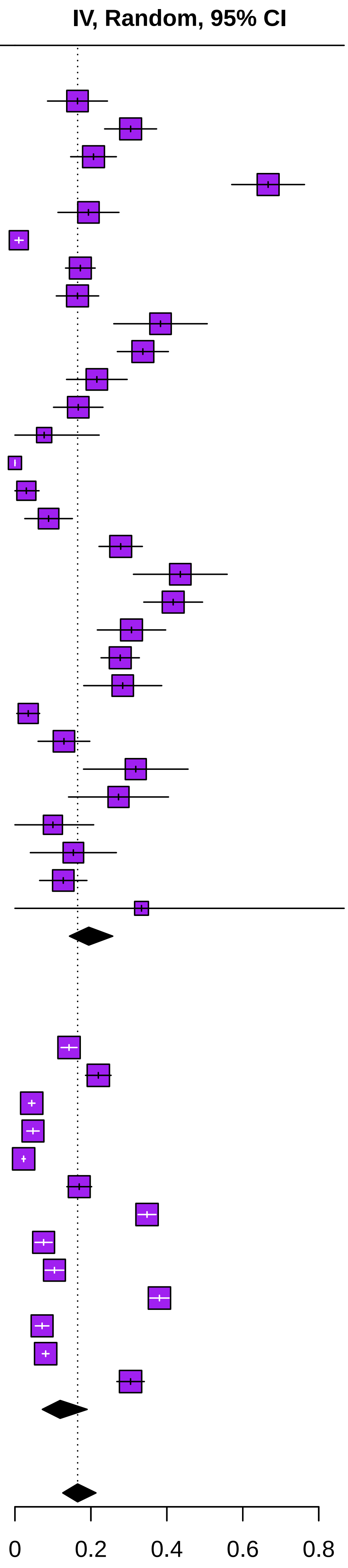

Meta-Analysis of Taste and Smell Dysfunction: Forest Plot by Sample Size Subgroups
